# Quality Characteristics and Volatile Components of Chili Oil Prepared from the Combination of Shuanla and Erjingtiao Peppers

**DOI:** 10.3390/molecules29235767

**Published:** 2024-12-06

**Authors:** Fang Yang, Simin Yao, Haibin Yuan, Can Yuan, Hongfeng Jia

**Affiliations:** 1 College of Culinary and Food Science Engineering, Sichuan Tourism University, Chengdu 610100, China; 18383515088@163.com (S.Y.); yuancan2019@sctu.edu.cn (C.Y.); 2 Faculty of Food and Biological Engineering, Chengdu University, Chengdu 610106, China; yuanhaibin@stu.cdu.edu.cn; 3 Cuisine Science Key Laboratory of Sichuan Province, Sichuan Tourism University, Chengdu 610100, China

**Keywords:** chili oil, capsaicinoids, volatile organic compounds, gas chromatography–ion mobility spectrometry, orthogonal partial least squares-discriminant analysis

## Abstract

This study aimed to investigate the influence of varying weight ratios of Shuanla and Erjingtiao peppers (10:0, 8:2, 6:4, 5:5, 4:6, 2:8, and 0:10, corresponding to samples PA, PB, PC, PD, PE, PF, and PG, respectively) on the sensory attributes, chromatism, acid values (AVs), peroxide values (POVs), capsaicinoids, and volatile organic compounds (VOCs) of seven chili oil samples. GC–IMS was employed to detect the VOCs of the chili oil samples, which were subsequently analyzed using multivariate statistical methods. The results revealed significant differences in pungency among the samples, with the PA sample exhibiting the strongest pungency. The PG sample demonstrated the highest values for a*, b*, and C*, while the PA sample displayed the highest L* and h*. The AVs of seven samples ranged from 0.490 ± 0.005 to 1.727 ± 0.015 mg/g. The POVs of the chili oil samples, ranging from 0.094 ± 0.000 to 0.127 ± 0.002 g/100 g, were significantly lower than those of extra virgin olive oil, 0.183 ± 0.001 g/100 g. The contents of capsaicinoids ranged from 15.26 ± 0.07 g/kg in the PA sample to 0.38 ± 0.00 g/kg in the PG sample (*p* < 0.05). Additionally, 56 volatile flavor substances were identified, and 10 key flavor compounds (ROAV ≥ 1) were screened among them. Multivariate data analysis via OPLS-DA indicated that 20 VOCs (VIP > 1) could serve as flavor markers in a clustering heat map to differentiate among the seven chili oil varieties. The findings of this study provide a valuable reference for the promotion of Shuanla and Erjingtiao peppers in chili oil production and the development of specific flavor profiles in chili oil to cater to diverse consumer preferences.

## 1. Introduction

Chili pepper, an annual or perennial plant belonging to the Solanaceae family, is characterized by its richness in capsaicin, capsanthin, vitamin C, polyphenols, flavonoids, and other substances [1,2,3]. These constituents confer various biological activities, including antioxidant effects, free radical scavenging, and anti-tumor properties [4]. Due to its palatable flavor and nutritional benefits, chili pepper is extensively utilized in culinary applications and the industrial production of hot sauces [5]. *Capsicum frutescens* L. and *Capsicum annuum* L. are the two primary cultivated species of the genus *Capsicum* in China, which encompass numerous varieties. Among these, Shuanla (*Capsicum frutescens* L. cv. Shuanlaense) is acknowledged as the hottest pepper variety in China, exhibiting higher levels of capsaicin, dihydrocapsaicin, and pungency compared to other varieties [6]. Erjingtiao (*Capsicum annuum* L.) is favored for its moderate spiciness, rich flavor, and vibrant red color when cooked. It is widely used in Pixian Doubanjiang (PXDBJ), a traditional Chinese condiment that enhances the spiciness, sauce aroma, and visual appeal of Sichuan cuisine [7].

Chili oil, a derivative of chili peppers, is a seasoning oil characterized by its unique flavor, prepared by infusing dried chili peppers in vegetable oil. It is noted for its strong aroma and moderate spiciness, playing an essential role in food preparation and enjoying widespread popularity [8]. The market offers a diverse range of chili peppers, with the genus *Capsicum* comprising over 200 varieties, each differing significantly in size, shape, flavor, and sensory attributes [9]. Li et al. demonstrated that the variety of chili peppers significantly influences the chroma, capsaicinoids, and primary flavor profiles of chili oil [10]. Zhu et al. investigated the sensory characteristics and pungency of seven different varieties of chili oils and chili powders, revealing that Indian pepper was characterized by its highest SHU rating of 85,909, a vibrant red color, a pronounced peppery flavor, and notable bitterness, while other samples displayed a broader range of flavors and milder textures [11]. Caporaso et al. demonstrated that infusing olive oil with varying concentrations of dried chili peppers significantly enhanced the oil’s antioxidant capacity and modified its volatile profile [12]. Currently, most analyses focus on chili oil produced from single varieties of chili peppers. However, as a popular condiment, chili oil is appreciated across various living environments and dietary cultures, which can influence consumer preferences. To address the diverse sensory preferences of consumers, the production of chili oil should capitalize on the unique qualities of various chili pepper types. This involves the precise control of flavor characteristics and oil quality through the strategic blending of different raw materials. Nevertheless, comprehensive analyses of the quality characteristics and volatile organic compounds (VOCs) of chili oil derived from mixtures of various chili pepper varieties are rare.

Gas chromatography–ion mobility spectrometry (GC–IMS) has emerged as a valuable technique for characterizing VOCs in food due to its excellent separation capabilities and high sensitivity. This method separates trace gases in the gas phase and characterizes chemical ionic substances based on their mobility rates in an electric field [13]. Principal component analysis (PCA) and orthogonal partial least squares-discriminant analysis (OPLS-DA) are powerful statistical modeling tools frequently employed to analyze VOC data. Hu et al. investigated the effects of vacuum sugaring osmosis and atmospheric pressure sugaring osmosis on the VOCs of dried fruits, and the volatile profiles of kumquats subjected to various treatments were effectively distinguished using GC–IMS combined with PCA [14]. Liu et al. identified a total of 56 VOCs in Tibetan black mutton with HS-GC–IMS, and flavor marker compounds, including 2-hexen-1-ol, 1-pentanol, octanal, etc., were highlighted by OPLS-DA [15]. Liu et al. classified and characterized sorghum using GC–IMS in conjunction with OPLS-DA to address the challenges posed by cultivar combinations during practical harvests. A total of 32 and 19 VOCs were selected to develop optimal classification models for stored and fresh sorghum types, respectively, with prediction accuracy exceeding 95% [16].

The primary objective of this present study was to compare the effects of varying ratios of Shuanla and Erjingtiao peppers on the physicochemical properties and flavor substances of chili oils. The volatile fingerprint spectra of these chili oils were established using GC–IMS, and chemometric tools such as PCA and OPLS-DA were employed to analyze the non-targeted volatile dataset, facilitating the identification of potential volatile markers for distinguishing between different chili oils. The findings may contribute to the promotion of Shuanla and Erjingtiao peppers in chili oil production and enhance existing production processes, particularly in the development of richly sensory chili oil products.

## 2. Results and Discussion

### 2.1. Sensory Analysis

**Figure 1:**
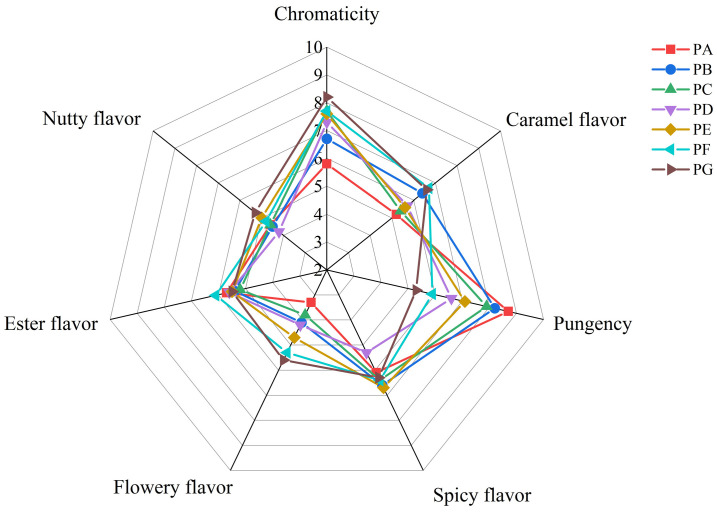
Sensory attribute intensity of chili oil samples.

The sensory evaluation was conducted on chili oil samples prepared from a combination of Shuanla and Erjingtiao peppers in various weight ratios: 10:0, 8:2, 6:4, 5:5, 4:6, 2:8, and 0:10, corresponding to samples PA, PB, PC, PD, PE, PF, and PG, respectively. The results are presented in Figure 1 and Table 1.

Significant differences were observed in pungency, with the PA sample exhibiting the highest level of pungency. In contrast, regarding the intensity of chromaticity, flowery flavor, and nutty flavor, the PG sample displayed the most pronounced levels. No significant differences were noted in ester flavor and spicy flavor; however, PA demonstrated the least intensity in caramel flavor.

The sensory evaluation scores for chili oil samples with varying ratios of Shuanla and Erjingtiao are tabulated in Table 1. As the proportion of Erjingtiao increased, the color of the chili oil was gradually accepted by panelists and significantly enhanced the flowery flavor of the chili oil samples (*p* < 0.05). A high ratio of Shuanla intensified the pungency taste of the chili oil, attributable to the significantly greater pungency of Shuanla compared to other varieties of chili peppers [6]. The observed decrease in color score with a reduced proportion of Erjingtiao can be attributed to the bright red color characteristic of Erjingtiao pepper.

### 2.2. Chromatism Analysis

The ΔE, L* (lightness), a* (redness), b* (yellowness), C*, and h* values for the various chili oil samples are presented in Table 2.

The L* value serves as an indicator of the clarity and glossiness of the oil; a higher L* value corresponds to clearer oil with improved gloss. There were no significant differences (*p* < 0.05) in the L* values among the seven chili oil samples, suggesting that their colors were clear and translucent. The colorimeter detected an increase in visible redness from PA to PG, reflected in the rising a* values. A higher b* value signifies a more pronounced yellow hue in the chili oil. The b* value for PG (13.55 ± 0.08) was greater than that of the other samples, with PA following closely at 13.30 ± 0.49. Interestingly, the b* value of chili oil prepared by mixing two types of paprika was lower than that of oil made from a single chili pepper. The C* value represents the saturation index of chili oil, while h* denotes a specific hue as defined on the chromaticity diagram [17]. The saturation C* values ranged from 14.80 ± 0.61 to 18.13 ± 0.06, with an increasing trend observed from samples PA to PG. The sample PG exhibited the highest C* value, which was significantly different from the five other samples (*p* < 0.05), except for PF. A larger h* value indicates a warmer hue, and vice versa. The h* values of the seven samples ranged from 51.12 ± 0.49 to 57.10 ± 0.89, with PA showing the largest h* value, significantly differing from the other samples (*p* < 0.05). This difference may be closely related to the warm red color characteristic of Shuanla. In terms of total color difference (ΔE), all chili oil samples showed considerable variation compared to extra virgin olive oil, with the PE sample exhibiting the most significant difference. Additionally, the ΔE value of sample PA was significantly different from that of the other samples (*p* < 0.05). In summary, the application of varying ratios of Shuanla and Erjingtiao in the preparation process resulted in discernible differences in the color of the chili oil samples. This variation can primarily be attributed to the carotenoid content of the raw chili pepper materials [18,19].

### 2.3. Acid Values

As an important reference parameter for assessing the conservation quality of fats and oils [20], the acid values (AVs) of chili oil samples are shown in Table 3. Significant differences (*p* < 0.05) were observed among the seven samples. The AVs of the seven samples were ranked as follows: PA > PB > PC > PD > PE > PF > PG, with values ranging from 0.490 ± 0.005 to 1.727 ± 0.153 mg/g. Additionally, the AVs of all seven chili oil samples exceeded that of extra virgin olive oil. This phenomenon can primarily be attributed to the extraction of organic acids from dried chili peppers by the olive oil [12]. Additionally, it may also result from the hydrolysis of the oil, the degradation of secondary oxidation products generated during thermal extraction, and the presence of oxygen or moisture in the air, all of which influence acidity and elevate the free fatty acid (FFA) value [21].

### 2.4. Peroxide Values (POVs)

The peroxide value (POV) is a classical method for assessing the degree of oxidation in edible oils, specifically measuring the formation of hydroperoxides [22]. The POVs of chili oil samples and extra virgin olive oil are shown in Table 3. The results indicated that the POV of sample PC was the lowest (0.094 g/100 g), and there was no significant difference (*p* < 0.05) among samples PA, PD, and PG, nor between the samples PE and PF. In addition, the POVs of the chili oil samples, which ranged from 0.094 ± 0.000 to 0.127 ± 0.002 g/100 g, were significantly lower than that of extra virgin olive oil, 0.183 ± 0.001 g/100 g, attributed to the antioxidant capacity of carotenoid and capsanthin [23]. This is consistent with the conclusion of Caporaso’s research [12]. The incorporation of chili pepper powder into extra virgin olive oil has been shown to enhance its shelf life. A threshold effect was observed, indicating a non-linear relationship between the quantity of chili pepper powder and the oil’s stability [24].

### 2.5. Capsaicinoids and Pungency

The characteristic pungency of chili oil is primarily attributed to the presence of capsaicinoids, with dihydrocapsaicin and capsaicin being the principal components [8]. Capsaicinoids are naturally occurring alkaloids found exclusively in *Capsicum* species. Along with certain vitamins and phenolic compounds, they contribute to a range of health benefits associated with chili peppers [25]. Sensory evaluations indicate that capsaicinoids are responsible for the sensations of heat and spiciness, characteristics that are highly valued by various consumer groups. Consequently, both chili peppers and chili oil have gained increasing significance in the food industry [26]. Currently, high performance liquid chromatography (HPLC) is the most reliable and efficient method for identifying and quantifying capsaicinoids, the results of which can be converted into SHU. The SHU serves as a critical indicator of pungency; a higher SHU indicates a hotter flavor in chili oil.

As shown in Table 4, significantly, the spiciness of the PA sample was the highest, while the PG sample exhibited the lowest spiciness, attributed to the Shuanla variety being the hottest pepper in China, with a higher SHU than other varieties [6]. The SHU values among the samples from PA to PG were not isolated; rather, they demonstrated a significant positive correlation. Specifically, samples with a higher proportion of Shuanla in the chili oil raw materials showed an increased content of capsaicinoids. This finding serves as a reference for producing chili oil products with varying levels of spiciness to cater to different consumer groups.

In comparison to the Erjingtiao chili oil prepared by Zhu et al. [11], which involved 24 h of extraction at 180 °C, our study produced Erjingtiao chili oil (PG sample) by mixing oil and chili pepper powder at 180 °C, followed by natural cooling over a period of 24 h. This method resulted in a higher total capsaicinoid content in the chili oil. Its advantage lies in the avoidance of prolonged heating, which can lead to an increase in peroxides [21] and the thermal degradation of capsaicin and dihydrocapsaicin through the cleavage of the alkyl group bonded to the amide [27,28]. Therefore, it is evident that the preparation method employed in our study is more effective in preserving the freshness of the chili oil and enhancing its stability.

### 2.6. Analysis of Volatile Flavor Compounds

#### 2.6.1. GC–IMS

**Figure 2:**
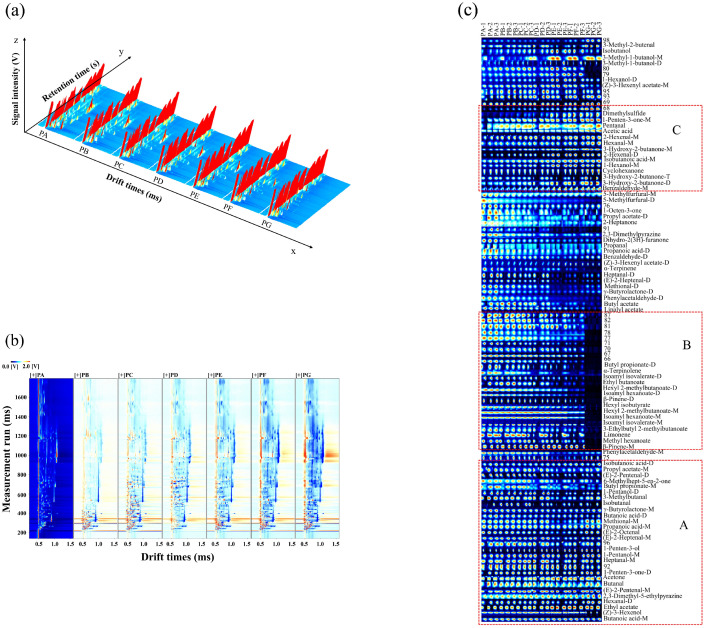
Three-dimensional topographic plot of VOCs (**a**), 2D topographic plot of VOCs (**b**), fingerprint spectra in the gallery plot (**c**). The measurements were taken in triplicate.

The volatile flavor substances were recovered and determined by GC–IMS. The data are presented as a 3D topographical visualization in Figure 2a. Each peak in the reaction ion peak (RIP) corresponds to a volatile flavor compound, with peak height serving as a quantitative measure. Although the VOCs in different chili oils are quite similar, their signal intensities vary slightly. In the two-dimensional plot, shown in Figure 2b, each point adjacent to the RIP represents a distinct VOC. The differential contrast model was employed to analyze the differences among the samples, with the PA sample serving as the reference. The spectra of the other chili oil samples were adjusted by subtracting the reference, resulting in a background that appeared white, indicating that the VOCs were consistent across the samples. Red spots signify that the substance content exceeds that of the reference, while blue spots indicate lower content compared to the reference [29]. In comparison to PA, the blue and red points exhibited an increasing trend from the PB sample to the PG sample, particularly within the retention time range of 900 to 1400 s. The VOCs in chili oil samples prepared with Shuanla demonstrated greater diversity, which diminished as the proportion of Shuanla decreased.

Although topographic plots can illustrate trends in the variation of VOCs, accurately assessing the dense substances represented on the spectrum remains challenging. The use of fingerprint spectra effectively addresses this issue, as demonstrated in Figure 2c. The color in the figure indicates the concentration of VOCs: brighter colors correspond to higher concentrations. As listed in Supplementary Table S1, it was observed that varying concentrations of individual compounds can generate multiple signals (dimers and trimers) that exhibit similar retention times but increased drift times due to their proton affinity and higher content [30,31].

A total of 56 VOCs were identified across the seven samples, derived from 102 signal peaks using the built-in NIST database (version 2020) and IMS database (version 2024) of GC–IMS. These compounds included six alcohols, 15 aldehydes, eight ketones, four carboxylic acids, 15 esters, three heterocycles, four terpenes, and one thioether. The VOCs of each chili oil sample, measured in triplicate, exhibited excellent reproducibility, while significant differences in VOCs were observed among the seven samples. In Region A, the aroma compounds, including butanoic acid-M, (Z)-3-hexenol, ethyl acetate, 2,3-dimethyl-5-ethylpyrazine, (E)-2-pentenal-M, butanal, acetone, heptanal-M, 1-pentanol-M, 1-penten-3-ol, (E)-2-heptenal-M, (E)-2-octenal, propanoic acid-M, methional-M, γ-butyrolactone-M, etc., were present in all seven chili oil samples, each exhibiting relatively high concentrations that imparted a shared flavor profile to the samples. In Region B, most of the volatile aroma compounds, such as α-terpinolene, ethyl butanoate, hexyl isobutyrate, hexyl 2-methylbutanoate-M, isoamyl hexanoate-M, isoamyl isovalerate-M, 3-ethylbutyl 2-methyibutanoate, limonene, methyl hexanoate, and β-pinene-M, were exclusively found in chili oil samples containing Shuanla as a raw material. Their concentrations decreased as the proportion of Shuanla in the raw materials diminished. Conversely, in Region C, the concentration of aromatic compounds, such as dimethylsulfide, 1-penten-3-one-M, pentanal, acetic acid, 2-hexenal-M, hexanal-M, 3-hydroxy-2-butanone, isobutanoic acid-M, 1-hexanol-M, cyclohexanone, 3-Hydroxy-2-butanone-M, and benzaldehyde-M, increased with the rising proportion of Erjingtiao in the raw materials.

**Figure 3:**
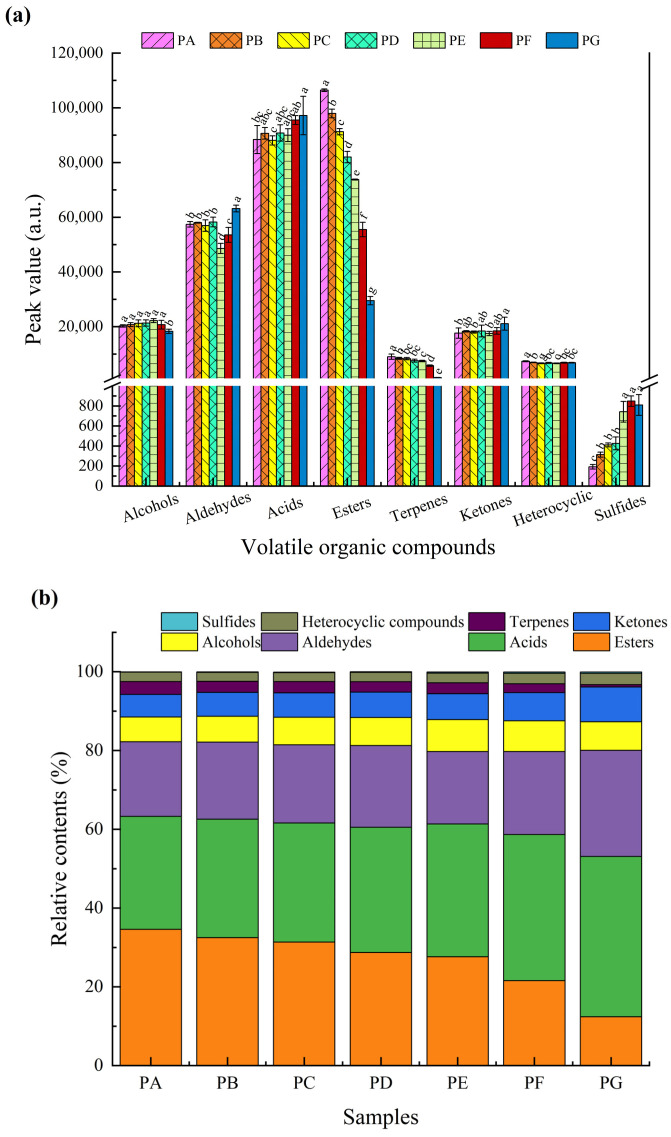
The peak value of VOCs (**a**) and relative content of VOCs (**b**) in chili oil samples. The different letters show a significant difference between chili oil samples in a relative amount of VOCs according to Duncan’s multiple range test (*p* < 0.05).

The peak value and relative percentage content of various identified VOCs are illustrated in Table 5 and Figure 3. Carboxylic acids exhibit the highest concentration, ranging from (28.70 ± 1.06)% to (40.74 ± 2.08)%, followed by esters and aldehydes, which range from (12.37 ± 0.53)% to (34.58 ± 0.61)% and (18.95 ± 0.18)% to (26.94 ± 0.65)%, respectively.

Only four acids were found in the chili oil samples: butyric acid, propionic acid, isobutyric acid, and acetic acid, with acetic acid comprising 46.70% to 58.04% of the total acids. These acids primarily originate from the release of compounds in paprika during the extraction process, with the high concentration of acetic acid likely resulting from the fermentation of chili pepper fruits during drying [12]. Acetic acid and propanoic acid produced acidic and vinegar-like aromas, while butyric acid and isobutyric acid imparted rancid, cheesy, and sweaty odors [32].

Esters were identified as the second most prevalent group of VOCs in the chili oil samples. Compared to other chili oil samples, PA exhibited a higher total ester content. Ethyl acetate, γ-butyrolactone-M, and propyl acetate-M were found in relatively high concentrations across all chili oil samples. Methyl hexanoate, 3-ethylbutyl 2-methyibutanoate, isoamyl isovalerate-M, isoamyl isovalerate-D, isoamyl hexanoate-M, isoamyl hexanoate-D, hexyl 2-methylbutanoate-M, hexyl 2-methylbutanoate-D, hexyl isobutyrate, and ethyl butanoate were detected in chili oil prepared from paprika with Shuanla, while their concentrations in the PG sample were notably minimal. Most ester compounds are characterized by fruity and floral aromas, primarily synthesized through the esterification of alcohols and FFAs or via the transesterification of esters [10].

Aldehyde compounds primarily originate from the oxidation of fats and contribute spiciness and fruity and floral aromas [33], thereby enhancing the overall sensory experience of chili oil. These compounds exhibit high diversity, elevated concentrations, and low threshold values, which significantly influence the flavor profile of chili oil. Notably, butanal, heptanal-M, (E)-2-heptenal-M, and (E)-2-octenal are found in high concentrations across various chili oil samples, imparting a common aroma to these products. The relative percentages of benzaldehyde-M, hexanal-M, 2-hexenal-M, and pentanal increase with a higher proportion of Erjingtiao in the chili powder raw material. Conversely, phenylacetaldehyde-M is predominantly present in the PA, PB, PC, and PD samples, which have a higher proportion of Shuanla in the paprika raw material.

Ketones are significant flavor compounds in the chili oil. Among the seven samples analyzed, the PG sample exhibited the highest ketone content at (8.84 ± 0.85)%, while the PA sample had the lowest at (5.73 ± 0.49)%. This variation indicates a trend where ketone levels increase with a higher proportion of Erjingtiao in the paprika raw material. It has been reported that ketones primarily arise from the oxidative decomposition of fats. Specifically, the oxidative decomposition of saturated fatty acids (SFAs), along with the further oxidation of olefins and alcohols generated from the breakdown of unsaturated fatty acids (UFAs), may serve as potential sources of ketone compounds [34]. Ketones typically present floral and fruity aromas. For instance, cyclohexanone, which has a minty fragrance, and 3-hydroxy-2-butanone, characterized by a creamy aroma, are found in the highest concentrations in the PG sample. Additionally, 1-octen-3-one, which imparts a fresh mushroom aroma; 2-heptanone, known for its fruity scent; and 6-methylhept-5-en-2-one, which has a lemongrass fragrance, are significantly more abundant in PA samples compared to the other samples.

Overall, the level of alcohol in PE was the highest among the samples, followed by PF. The characteristic alcohols identified in PE and PF include 1-hexanol-M, 1-hexanol-D, and isobutanol. Both 1-hexanol-M and 1-hexanol-D exhibit a fruity aroma with subtle hints of green leaf, while isobutanol is characterized by a faint oily odor and a narcotic-like flavor. Alcohol arises as a byproduct of lipid oxidation, primarily generated through the oxidative degradation of UFAs [35].

Terpenes represent a significant class of aromatic compounds. Among the identified VOCs, only four terpenes were detected. α-terpinolene, β-pinene-M, and β-pinene-D, contribute pine and resinous aromas to chili oil, while limonene and α-terpinene impart citrus and lemon-like fragrances to the chili oil. The total levels of terpenes in the VOCs of the chili oil samples exhibit a declining trend as the proportion of Erjingtiao in the raw materials increases, with a relative percentage content ranging from (3.26 ± 0.39)% to (0.59 ± 0.02)% from PA to PG.

Despite the relatively low concentrations of heterocyclic and thioether compounds, their contribution to the overall flavor is significant due to their low flavor thresholds. Heterocyclic compounds generated from the Maillard reaction, such as 2,3-dimethyl-5-ethylpyrazine, 2,3-dimethylpyrazine, 5-methylfurfural-M, and 5-methylfurfural-D, contribute to the pleasant roasted flavor in chili oil [36]. Dimethyl sulfide, which possesses the characteristic taste and aroma of cooked celery, was formed from methionine Strecker degradation, which yields methional, which decomposes into methanethiol, subsequently forming dimethyl sulfide [37,38].

#### 2.6.2. Key Volatile Aroma Compounds

In comparison to the relative content of VOCs, the relative odor activity value (ROAV) offers a more precise representation of the contribution of VOCs to the overall aroma profile of chili oil samples. The ROAV of methional-M, a significant contributor to the chili oil samples’ overall flavor profile, was standardized at 100. VOCs exhibiting a ROAV of 1 or greater are deemed pivotal in defining the aroma of chili oil. Compounds with a ROAV between 0.1 and 1, while not dominant, significantly influence the flavor profile. Conversely, VOCs with a ROAV below 0.1 contribute negligibly to the oil’s flavor.

As shown in Table 6, VOCs contributing to the aroma of chili oil were selected based on their ROAV, accompanied by sensory descriptions. A total of 10 key VOCs (ROAV ≥ 1) and 23 modifying substances (0.1 < ROAV < 1) were detected among the samples. Specifically, the PB, PC, PD, and PF samples each contained nine key VOCs, while the PA, PF, and PG samples contained eight, nine, and seven key VOCs, respectively. Notably, isobutyraldehyde was identified as a flavor-modifying substance (ROAV < 1) in both PA and PF. However, it exhibited a ROAV greater than 1 in PB, PC, PD, PE, and PF, indicating its role as a key flavor compound that imparts fresh, floral, and pungent notes. 3-Methylbutanal was recognized as a key flavor compound in PA, PB, PC, PD, and PE, primarily contributing to aromas reminiscent of peach, sourness, and chocolate. Conversely, acetic acid was identified as a key flavor compound solely in PE and PF. The key VOCs shared across all samples included methional-M, methional-D, 1-penten-3-one-D, 1-penten-3-one-M, (E)-2-octenal, dimethylsulfide, benzaldehyde-M, butanoic acid-M, and phenylacetaldehyde-M. Among these, methional-M, known for imparting a strong cooked potato aroma, was found to have the most significant impact on the flavor of the chili oil samples, with a ROAV defined as 100. The ROAV of (E)-2-octenal ranged from 11.08 ± 0.09 to 14.30 ± 0.69, primarily contributing to aromas of fats and nuts. The ROAV of 1-penten-3-one-M varied from 8.56 ± 4.23 to 24.01 ± 1.31, while that of 1-penten-3-one-D ranged from 22.75 ± 11.72 to 48.17 ± 0.49, with both compounds predominantly imparting mustard, garlic, and onion notes. The ROAV of dimethyl sulfide ranged from 1.50 ± 0.18 to 11.39 ± 1.04, indicating significant variability in its contribution to the flavor profiles of chili oil samples; however, all samples demonstrated notable effects. Phenylacetaldehyde-M contributed aromas of hyacinth, honey, and clover, with a ROAV ranging from 1.36 ± 0.02 to 2.51 ± 0.07 across all samples. Benzaldehyde-M served as a key flavor substance, primarily characterized by a bitter almond aroma, with the highest ROAV recorded in PE (4.34 ± 0.18) and the lowest in PA (1.59 ± 0.02).

#### 2.6.3. Principal Component Analysis

PCA is a multivariate statistical method that utilizes multiple variables to perform linear transformations. This technique selects a limited number of significant variables and, through dimensionality reduction, extracts key features for linear analysis while preserving essential information within a few uncorrelated principal components. To further elucidate the differences and similarities among chili oil samples, PCA was applied to the quantitative results of the VOCs directly through the Dynamic PCA plugin (version 1.4.1) built-in FlavourSpec^®^ instrument (G.A.S., Dortmund, Germany).

**Figure 4:**
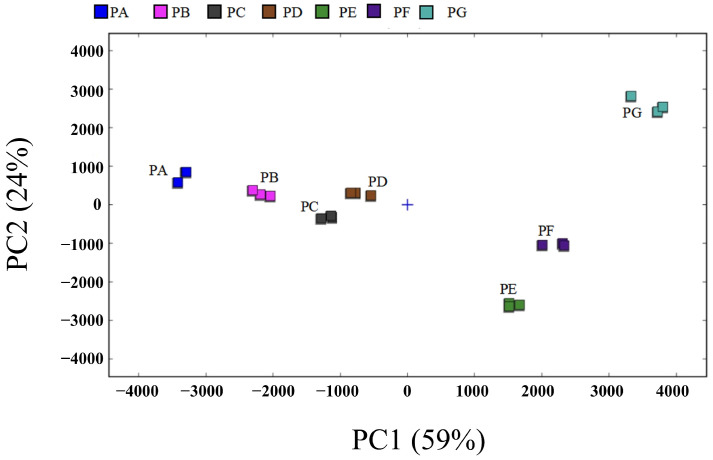
PCA of the chili oil samples. The GC–IMS analysis was taken in triplicate for all the samples.

As shown in Figure 4, PC1 and PC2 accounted for 59% and 24% of the total variance, respectively. This indicates that the dimensionality reduction retains the majority of the relevant information regarding the VOCs, thereby reflecting the overall characteristics of the samples [39]. The contribution rate of PC1 is noticeably greater than that of PC2. A larger horizontal distance of samples along PC1 corresponds to more obvious flavor differences. The PE and PF samples were located in the fourth quadrant, while the PG sample was situated in the first quadrant. The PA, PB, and PD samples were found in the second quadrant, and the PC sample was positioned in the third quadrant. The distribution of the horizontal axis distances indicates that the aromas of PB, PC, and PD are relatively similar, whereas the distance distributions of the remaining samples exhibit significant differences. This indicates that there are noticeable differences in the aromas of the PE, PF, and PG samples. Furthermore, these findings indicate significant variations in the flavor characteristics of chili oil prepared with different ratios of Erjingtiao and Shuanla. Additionally, PCA effectively differentiates the VOCs among the various chili oil samples.

#### 2.6.4. Orthogonal Partial Least Squares-Discriminant Analysis

**Figure 5:**
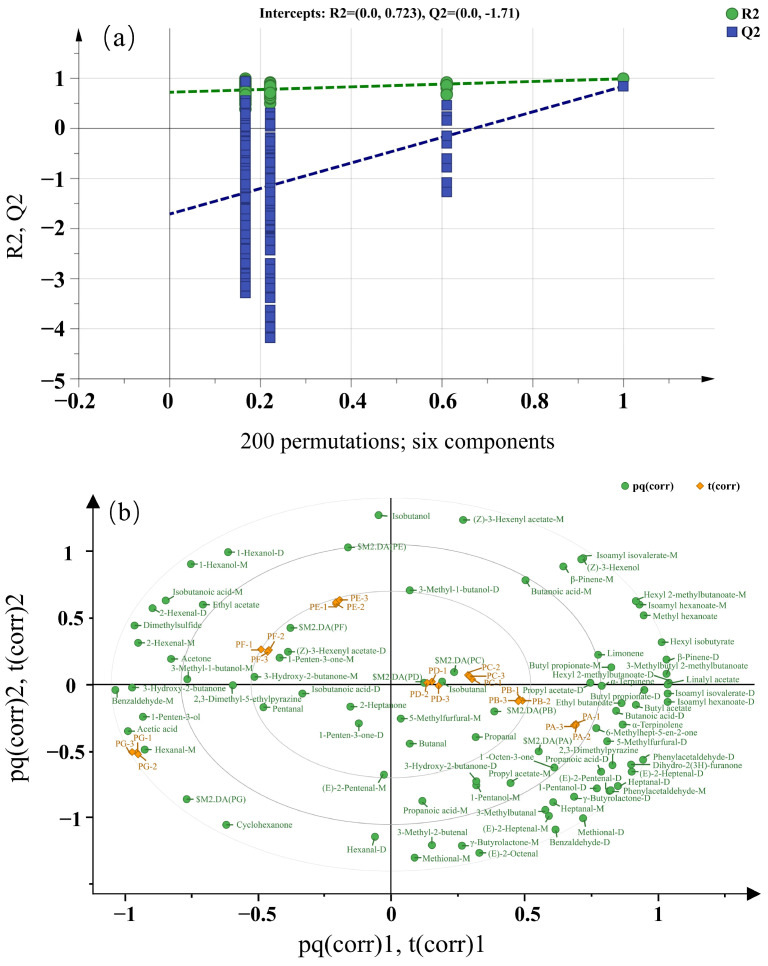
The cross-validation results (**a**) and biplot (**b**) of chili oil samples with OPLS-DA model. The green dashed line in image (a) represents the regression line fitted to the R2 point, while the blue dashed line corresponds to the regression line fitted to the Q2 point.

OPLS-DA is recognized for its superior discrimination capabilities [40]. To further examine the influence of VOCs on the variations among the samples, an OPLS-DA model analysis was performed on the VOCs present in the chili oil samples. The fit indices R2X, R2Y, and Q2 are 0.996, 0.981, and 0.696, respectively. Here, R2X denotes the independent variable, R2Y denotes the dependent variable, and Q2 indicates the model’s predictive ability. These values indicate that the model exhibits strong predictive validity [16]. Model validation was carried out through 200 permutation tests. As illustrated in Figure 5a, the intersection point of the Q2 regression line with the vertical axis is negative, suggesting that the model does not exhibit overfitting and that the validation process was effective. Consequently, the results obtained can be utilized to differentiate the characteristic substances of various chili oil varieties.

As illustrated in Figure 5b, all chili oil samples could be distinctly differentiated from one another. The PC and PD samples were concentrated along the midline of the first and fourth quadrants, suggesting a similarity in flavor between these two samples. PA and PB were located in the fourth quadrant, with the substances surrounding PA being significantly more abundant than those in the other samples. PE and PF were positioned in the second quadrant, while PG was located in the third quadrant, indicating that the flavor profiles of these samples differ significantly from those of the others. The flavor attributes of each chili oil sample were not influenced by a single volatile compound but rather were shaped by the balance and interactions among multiple VOCs. Consistent with PCA, the biplot of OPLS-DA could effectively distinguish the influence of VOCs among the seven chili oil samples.

The strength and impact of each flavor compound in identifying different varieties of chili oil samples were determined by the calculation of the variable importance in projection (VIP) value. A total of 20 VOCs (VIP > 1) were identified, including butanoic acid-D, butanoic acid-M, hexyl 2-methylbutanoate-D, hexyl 2-methylbutanoate-M, acetic acid, isoamyl hexanoate-D, isoamyl hexanoate-M, isoamyl isovalerate-D, isoamyl isovalerate-M, linalyl acetate, benzaldehyde-M, 2-hexenal-D, (Z)-3-hexenol, phenylacetaldehyde-M, ethyl acetate, β-pinene-M, isobutanoic acid-D, hexyl isobutyrate, 1-penten-3-one-D, (E)-2-octenal, acetone, γ-butyrolactone-M, ethyl butanoate, and 1-pentanol-D; some of which were present in both monomeric and dimeric forms. These compounds were considered to significantly contribute to the differentiation among the samples.

**Figure 6:**
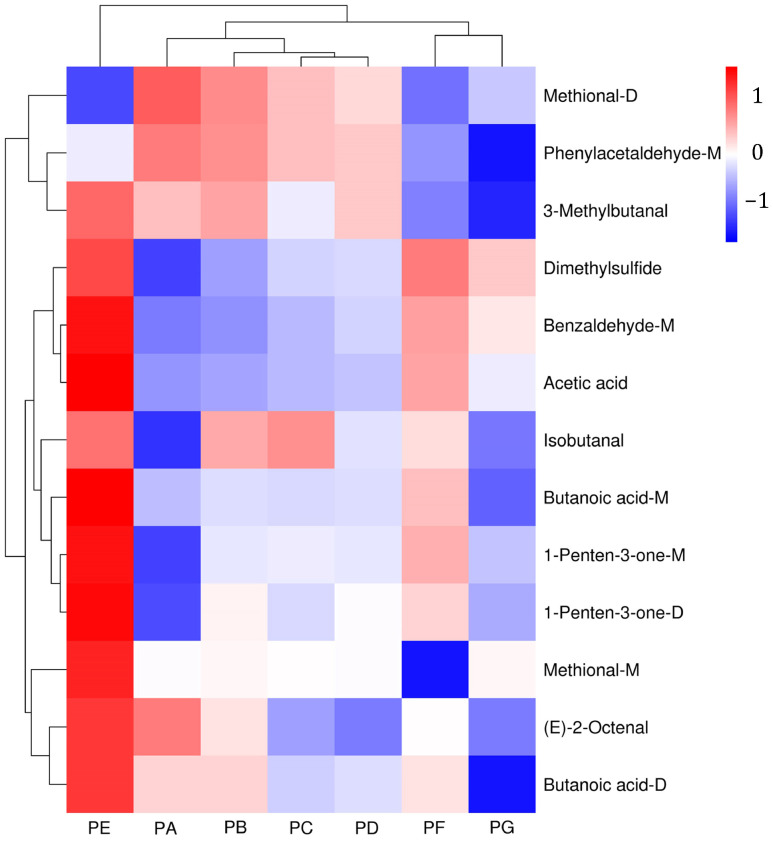
Clustering heat map of VOCs (VIP > 1) of chili oil samples.

A clustering heatmap was generated based on the VOCs (VIP > 1) to effectively and clearly visualize the differences among the chili oil samples. As illustrated in Figure 6, the heatmap presents the average profile of each volatile compound for differentiation, employing a color scale with hierarchical clustering analysis (HCA) [41]. Distinct colors indicate the concentration levels of each volatile compound. Together with column clustering, the chili oil samples were distinctly classified, correlating with previous PCA results and indicating that varying ratios of Erjingtiao and Shuanla significantly influence the VOCs of the chili oil samples.

## 3. Materials and Methods

### 3.1. Main Materials and Reagents

The dried chili peppers of Shuanla and Erjingtiao were purchased from a local market in Chengdu city (China). Extra virgin olive oil was purchased from Shandong Luhua Group Co., Ltd. (Laiyang, China). Refined edible salt was purchased from Sichuan Salt Industry Corporation (Chengdu, China).

Main reagents: glacial acetic acid (99.5% purity), dichloromethane (≥ 99.5% purity), sodium thiosulfate (99% purity), potassium iodide (99% purity), soluble starch (99% purity), and potassium iodate (99% purity) were purchased from Chengdu Cologne Chemical Co., Ltd. (Chengdu, China).

### 3.2. Preparation of Chili Oil

**Figure 7:**
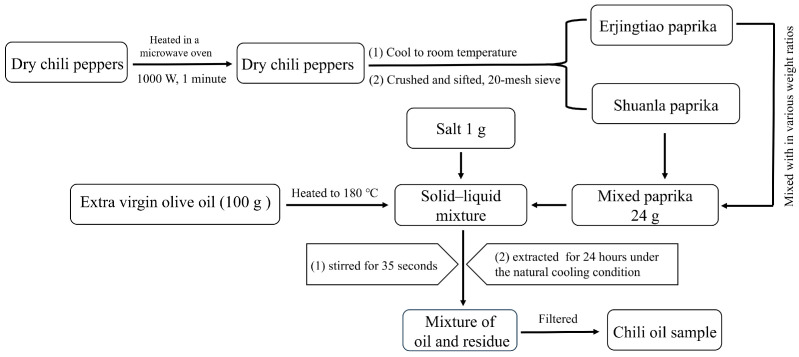
The scheme of preparation for chili oil samples.

The chili oil samples were prepared following the scheme in Figure 7. Dry chili peppers were heated in a microwave oven (1000 W) for 1 min and then allowed to cool to room temperature. Following this, they were crushed and sifted through a 20-mesh sieve. Shuanla and Erjingtiao powders were mixed in various weight ratios for the preparation of chili oil samples PA, PB, PC, PD, PE, PF, and PG, shown in Table 7. Seven portions of extra virgin olive oil (100 g each) were heated to 180 °C and then mixed with each mixture of powder (24 g) and salt (1 g). The resulting mixture was vigorously stirred for 35 s to ensure thorough incorporation. The extraction process continued for 24 h under natural cooling conditions. The upper clear oil from each sample was subsequently stored at 4 °C for further analysis.

### 3.3. Descriptive Sensory Analysis

A panel of ten judges, proficient in sensory evaluation, underwent training to assess chili oil using a descriptive analysis based on a structured nine-point scale (1: non-perceptible; 5: medium intensity; 9: extremely intense). A comprehensive list of descriptors was developed and implemented, with attributes further defined during sensory focus groups utilizing flash profile techniques. The evaluated attributes included chromaticity, caramel flavor, pungency, spicy flavor, flowery flavor, ester flavor, and nutty flavor. Evaluations of the chili oil were conducted in a sensory laboratory under white light. The panelists participated in three sessions, during which seven samples were evaluated at one time, resulting in a total of three replicates. The samples were evaluated uniformly, with the presentation order randomized among assessors. Chili oil samples (10 mL) were presented in transparent glass cups, coded randomly, and provided to the examiners in separate containers.

### 3.4. Chromatism Analysis

The L* (lightness), a* (redness), b* (yellowness), C*, and h* values of different chili oils were determined with a colorimeter (NH310, Shenzhen Sanenshi Technology Co., Ltd., Shenzhen, China). The C* value signifies the saturation index of chili oil, h* denotes a specific hue as defined on the chromaticity diagram, and ΔE represents the total color difference between chili oil sample and the extra olive oil [17]. The instrument was calibrated with a black and white ceramic tile before measurement. After calibration, each chili oil sample (10 g) was pipetted in a glass sample cell cuvette (volume, 25 mL). The measurements were taken in triplicate. The ΔE value was calculated with Equation (1).

(1) $$\Delta E=\sqrt{\left[{\left(L^{*}-{L^{*}}_{0}\right)}^{2}+{\left(a^{*}-{a^{*}}_{0}\right)}^{2}+{\left(b^{*}-{b^{*}}_{0}\right)}^{2}\right]}$$

where the subscript ‘0’ indicates the color of the extra virgin olive oil (reference sample), and the values of L*_0_, a*_0_, and b*_0_ are 66.58, 4.12, and 17.50, respectively.

### 3.5. Determination of Acid Values

The acid value is defined as the amount of potassium hydroxide, measured in milligrams, required to neutralize the free acids present in 1 g of chili oil. The concentration of FFAs in the oil was determined by titrating a methanol solution of the oil with a sodium hydroxide solution, using phenolphthalein as the indicator. The method employed was in accordance with AOCS Cd 3d-63 [42].

### 3.6. Determination of Peroxide Values (POVs)

Peroxide values (POVs) were determined by measuring the amount of iodine produced from the reaction between peroxides (derived from fats or oils) and iodide ions under acidic conditions. The POVs were assessed in accordance with AOCS Cd 8-53 method [43]. In this procedure, 2 g of the chili oil sample (accurate to 0.001 g) was weighed in an Erlenmeyer flask, followed by the addition of 30 mL of a glacial acetic acid–chloroform solvent (3:2). Then, the mixture was stirred. Subsequently, 1 mL of a saturated potassium iodide solution was introduced. The mixture was placed in a dark environment for 3 min. Afterward, 100 mL of distilled water and 1 mL of starch indicator were added, and the solvent was titrated with a 0.01 mol/L sodium thiosulfate solution.

### 3.7. Determination of Capsaicinoids and Pungency

The content of capsaicinoids was determined by HPLC (Agilent 1260, Agilent Technologies, Santa Clara, CA, USA). An aliquot of 2 g sample (accurate to 0.001 g) was mixed with 25 mL of a methanol–tetrahydrofuran solvent (*v*:*v* = 1:1) in a 100 mL beaker. The beaker was then sealed with plastic wrap and punctured with a few small holes using a needle. The mixture was extracted using an ultrasonic oscillator in a water bath at 60 °C for 30 min. Subsequently, the solution was filtered through filter paper, and the filtrate was collected. An additional 25 mL of the methanol–tetrahydrofuran solvent (*v*:*v* = 1:1) was added to the residue on the filter paper, followed by ultrasonic extraction for 10 min. This extraction process was repeated twice. The filtrates from the three extractions were combined and concentrated to a volume of 10 mL to 20 mL using a rotary evaporator at 70 °C. The concentrated solution was then diluted to a final volume of 50 mL with the methanol–tetrahydrofuran solvent (*v*:*v* = 1:1). Finally, the solution was filtered through a 0.45 µm filter membrane before being subjected to chromatographic analysis.

The HPLC condition parameters were applied as follows: Chromatographic column, Zorbax SB-C18 (4.6 mm × 250 mm, 5 μm, Agilent Technologies, Santa Clara, CA, USA). Methanol–water (*v*:*v* = 65:35) as mobile phase. Injection volume: 10 μL. The flow rate was 1 mL/min. UV detection wavelength: 280 nm. Column oven temperature: 30 °C.

To quantify capsaicinoids, calibration curves were established using external standards of capsaicin and dihydrocapsaicin, which were evenly distributed across a concentration range of 0–100 μg/mL. The calculation formula is presented in Equation (2):

(2) $$M=\frac{M_{a}+M_{b}}{0.9}$$

where M represents the concentration of capsaicinoids (g/kg); M_a_ denotes the concentration of capsaicin (g/kg); M_b_ indicates the concentration of dihydrocapsaicin (g/kg); 0.9 is the coefficient used to convert dihydrocapsaicin and capsaicin to capsaicinoids according to reference [11].

Pungency: the capsaicinoids content was converted to Scoville Heat Units (SHU) according to Formula (3):

(3) $$SHU=M\times 0.9\times (16.1\times 10^{3})+M\times 0.1\times (9.3\times 10^{3})$$

where M represents the concentration of capsaicinoids (g/kg). The conversion coefficient for capsaicin and dihydrocapsaicin is 0.9. The conversion factor for capsaicin or dihydrocapsaicin to SHU is 16.1 × 10^3^. The conversion coefficient for other capsaicinoids is 0.1, while the conversion factor for other capsaicinoids to SHU is 9.3 × 10^3^ according to reference [11].

### 3.8. GC–IMS Analysis

The VOCs of chili oil samples were detected by GC–IMS (FlavourSpec^®^, G.A.S., Dortmund, Germany) with MXT-WAX capillary column (30 m × 0.53 mm × 1 µm, Restek, Mount Ayr, IN, USA). The method developed by Zhang et al. [44] was adopted and enhanced for the analysis of HS-GC–IMS. Specifically, 1.5 g chili oil was weighed into a 20 mL headspace-glass sampling vial. The samples were incubated at 80 °C for 20 min, incubation speed 500 r/min. Following incubation, 500 µL of headspace was automatically injected into the injector under splitless injection mode with a syringe at 85 °C. The cleaning time was maintained at 30 s, and high–purity N_2_ (purity ≥ 9.99%) was used as the carrier gas.

GC conditions: Column temperature was set to 60 °C; the total running time was 40 min. The flow rate was initially 2 mL/min, maintained for 2 min, then increased to 10 mL/min over a duration of 10 min, and subsequently raised to 100 mL/min over the final 40 min.

IMS conditions: The column temperature was also maintained at 60 °C, with an analysis time of 40 min. The drift gas used was high–purity N_2_ (purity ≥ 9.99%).

The retention index (RI) of VOCs was calculated using ketones C4–C9 (2-butanone, 2-pentanone, 2-hexanone, 2-heptanone, 2-octanone, 2-nonanone) as external reference. The RI and ions’ drift times of VOCs were compared with those of the standards in the GC–IMS library to identify detected VOCs. The experiments of all samples were conducted in triplicate. The relative quantification of each VOC was determined based on its peak intensity. The 3D topographic plots, 2D difference plots, and gallery plots were constructed using VOCal software (version 0.2.9, G.A.S., Dortmund, Germany), which integrates the Reporter plugin (version 1.4.03), and Gallery plugin (version 1.2.6), supplemented by the FlavourSpec^®^ instrument (G.A.S., Dortmund, Germany).

### 3.9. Identification of Key VOCs

The key VOCs were screened and determined by ROAV method [45]. The ROAV of the component (stan) that contributes most significantly to the overall flavor of the sample is defined as 100. The ROAV of the other components (A) was calculated according to Equation (4).

(4) $${ROAV}_{A}\approx \frac{100\times C_{A}\times T_{\mathrm{stan}}}{C_{\mathrm{stan}}\times T_{A}}$$

where C_stan_ denotes the relative content of the component (stan); C_A_ represents the relative content of the other components (A); T_stan_ is the threshold of the component (stan); T_A_ is the threshold value of the other components (A). The threshold values in vegetable oil are documented in the literature [46].

### 3.10. Statistical Analysis

The GC–IMS spectra were analyzed using VOCal software (version 0.2.9), and the difference profiles and fingerprint spectra of VOCs were constructed with the Reporter plugin (version 1.4.03), and Gallery plugin (version 1.2.6) enabling comprehensive sample analysis from various perspectives. The experimental results are presented as mean ± standard deviation (SD) of triplicate analysis, calculated using IBM SPSS software (version 22.0, IBM Corp., Armonk, NY, USA). Data from the samples, measured using GC–IMS, underwent principal component analysis (PCA) directly through Dynamic PCA plugin (version 1.4.1). Graphing was performed using Origin 2021 (OriginLab Corporation, Northampton, MA, USA) software. Differences among samples were analyzed using ANOVA in SPSS (version 22.0), with the Duncan method applied for identifying significant differences (*p* < 0.05). Additionally, SIMCA 14.1 (MKS Umetrics, Umea, Sweden) was utilized for OPLS-DA, and the heat map cluster analysis were performed using BMKCloud https://www.biocloud.net (accessed on 8 October 2024).

## 4. Conclusions

This study analyzed the sensory attributes, chromatism, acid values, peroxide values, and capsaicinoids content of seven chili oil samples derived from varying ratios of Shuanla and Erjingtiao peppers. By employing GC–IMS technology, we provided a comprehensive assessment of the changes in volatile components. The results indicated that each chili oil sample exhibited unique characteristics in sensory attributes, chromatic properties, acid values, peroxide values, and capsaicinoids, contributing to their distinct flavors.

Furthermore, by integrating qualitative sensory descriptions with quantitative metrics such as color differences and pungency, we could more scientifically and objectively differentiate chili oils produced from varying ratios of Shuanla and Erjingtiao peppers. This study offers a foundational experimental basis and data support for the promotion of Shuanla and Erjingtiao peppers in chili oil production and the formulation of specific flavored pepper oils tailored to meet the preferences of diverse consumer groups. Additionally, the preparation process significantly affects the inherent flavor, which is subject to variation due to factors such as extraction temperature, extraction time, and the granularity of the chili particles. Consequently, it is imperative to investigate the evolution of key flavor compounds throughout the chili oil preparation process.

## Figures and Tables

**Table 1:** Mean scores of sensory attributes of chili oil samples.

Samples	Chromaticity	Caramel Flavor	Pungency	Spicy Flavor	Flowery Flavor	Ester Flavor	Nutty Flavor
PA	5.8 ± 0.6 ^d^	5.2 ± 0.8 ^c^	8.7 ± 0.5 ^a^	6.1 ± 1.2 ^b^	3.3 ± 0.7 ^e^	5.7 ± 0.7 ^ab^	4.6 ± 0.7 ^ab^
PB	6.7 ± 0.5 ^c^	6.4 ± 0.7 ^ab^	8.2 ± 0.6 ^ab^	6.6 ± 1.0 ^a^	4.1 ± 0.3 ^cde^	5.4 ± 0.5 ^ab^	4.5 ± 0.8 ^ab^
PC	7.7 ± 0.7 ^ab^	5.4 ± 0.5 ^c^	7.9 ± 0.6 ^b^	6.4 ± 0.7 ^a^	3.8 ± 0.8 ^de^	5.2 ± 0.6 ^b^	4.6 ± 0.8 ^ab^
PD	7.3 ± 0.7 ^bc^	5.7 ± 0.5 ^bc^	6.6 ± 0.7 ^c^	5.3 ± 0.5 ^b^	4.2 ± 0.8 ^cd^	5.6 ± 0.7 ^ab^	4.2 ± 1.1 ^b^
PE	7.6 ± 0.7 ^ab^	5.6 ± 0.5 ^c^	7.1 ± 0.3 ^c^	6.7 ± 0.7 ^a^	4.7 ± 0.8 ^bc^	5.5 ± 0.7 ^ab^	5.0 ± 0.7 ^ab^
PF	7.7 ± 0.5 ^ab^	6.7 ± 0.7 ^a^	5.9 ± 0.6 ^d^	6.4 ± 0.5 ^a^	5.3 ± 0.5 ^ab^	6.1 ± 0.7 ^a^	4.8 ± 0.9 ^ab^
PG	8.2 ± 0.4 ^a^	6.6 ± 0.7 ^a^	5.3 ± 0.5 ^d^	6.3 ± 0.5 ^a^	5.6 ± 1.0 ^a^	5.5 ± 0.8 ^ab^	5.3 ± 0.7 ^a^

The experimental results are presented as mean ± standard deviation of triplicate analysis. The different letters show a significant difference between chili oil samples in sensory attributes according to Duncan’s multiple range test (*p* < 0.05).

**Table 2:** Color space values of chili oil samples.

Samples	a*	b*	C*	L*	h*	△E
PA	8.60 ± 0.03 ^e^	13.30 ± 0.49 ^a^	15.83 ± 0.42 ^de^	63.61 ± 0.21 ^a^	57.10 ± 0.89 ^a^	6.83 ± 0.38 ^c^
PB	8.84 ± 0.16 ^e^	11.87 ± 0.64 ^b^	14.80 ± 0.61 ^f^	62.57 ± 0.78 ^a^	53.30 ± 1.06 ^b^	8.39 ± 0.73 ^b^
PC	9.65 ± 0.15 ^d^	11.97 ± 0.04 ^b^	15.37 ± 0.08 ^ef^	63.16 ± 0.07 ^a^	51.12 ± 0.49 ^bc^	8.54 ± 0.14 ^b^
PD	10.42 ± 0.40 ^c^	13.22 ± 0.71 ^a^	16.84 ± 0.80 ^bc^	63.29 ± 0.64 ^a^	51.74 ± 0.48 ^b^	8.33 ± 0.30 ^b^
PE	11.29 ± 0.49 ^b^	11.92 ± 0.87 ^b^	16.45 ± 0.45 ^cd^	62.24 ± 1.90 ^a^	46.54 ± 3.09 ^e^	10.14 ± 1.53 ^a^
PF	11.33 ± 0.18 ^b^	13.19 ± 0.37 ^a^	17.39 ± 0.40 ^ab^	63.49 ± 0.16 ^a^	49.31 ± 0.44 ^cd^	8.96 ± 0.04 ^ab^
PG	12.04 ± 0.01 ^a^	13.55 ± 0.08 ^a^	18.13 ± 0.06 ^a^	63.27 ± 0.14 ^a^	48.36 ± 0.14 ^de^	9.45 ± 0.07 ^ab^

The experimental results are presented as mean ± standard deviation of triplicate analysis. The different letters show a significant difference between chili oil samples in color space values according to Duncan’s multiple range test (*p* < 0.05).

**Table 3:** Acid values and peroxide values of chili oil samples.

Samples	Acid Values (mg/g)	Peroxide Values (g/100 g)
PA	1.727 ± 0.015 ^a^	0.126 ± 0.001 ^b^
PB	1.557 ± 0.015 ^b^	0.123 ± 0.000 ^c^
PC	1.177 ± 0.006 ^c^	0.094 ± 0.000 ^e^
PD	1.133 ± 0.021 ^d^	0.127 ± 0.002 ^b^
PE	0.810 ± 0.008 ^e^	0.118 ± 0.000 ^d^
PF	0.597 ± 0.003 ^f^	0.118 ± 0.000 ^d^
PG	0.490 ± 0.006 ^g^	0.126 ± 0.000 ^b^
Olive oil	0.303 ± 0.008 ^h^	0.183 ± 0.001 ^a^

The experimental results are presented as mean ± standard deviation of triplicate analysis. The different letters show a significant difference between chili oil samples in acid values and peroxide values according to Duncan’s multiple range test (*p* < 0.05).

**Table 4:** The content of capsaicinoids in chili oil samples.

Samples	Capsaicin Content (g/kg)	Dihydrocapsaicin Content (g/kg)	Capsaicinoids Content (g/kg)	Scoville Heat Unit (SHU)	Pungency Degree
PA	10.34 ± 0.06 ^a^	3.39 ± 0.01 ^a^	15.26 ± 0.07 ^a^	235,355.33 ± 1134.98 ^a^	1569.33 ± 7.51 ^a^
PB	8.06 ± 0.02 ^b^	2.68 ± 0.01 ^b^	11.93 ± 0.03 ^b^	184,032.67 ± 505.76 ^b^	1226.67 ± 3.21 ^b^
PC	6.54 ± 0.02 ^c^	2.17 ± 0.00 ^c^	9.68 ± 0.02 ^c^	149,188.67 ± 318.70 ^c^	994.33 ± 2.08 ^c^
PD	5.54 ± 0.06 ^d^	1.84 ± 0.02 ^d^	8.20 ± 0.08 ^d^	126,434.00 ± 1297.79 ^d^	842.67 ± 8.33 ^d^
PE	4.50 ± 0.04 ^e^	1.40 ± 0.01 ^e^	6.56 ± 0.05 ^e^	101,155.00 ± 766.41 ^e^	674.67 ± 5.03 ^e^
PF	2.22 ± 0.01 ^f^	0.74 ± 0.00 ^f^	3.29 ± 0.02 ^f^	50,706.00 ± 231.75 ^f^	338.00 ± 1.73 ^f^
PG	0.23 ± 0.00 ^g^	0.11 ± 0.00 ^g^	0.38 ± 0.00 ^g^	5803.33 ± 23.46 ^g^	39.00 ± 0.00 ^g^

The experimental results are presented as mean ± standard deviation of triplicate analysis. The different letters show a significant difference between chili oil samples according to Duncan’s multiple range test (*p* < 0.05).

**Table 5:** Relative amount (%) of VOCs identified by GC–IMS.

Compounds ^1^	Relative Amount (%)						
	PA	PB	PC	PD	PE	PF	PG
**Alcohols (6)**							
(Z)-3-Hexenol	2.12 ± 0.10 ^b^	2.15 ± 0.07 ^b^	2.30 ± 0.06 ^b^	2.34 ± 0.25 ^b^	2.71 ± 0.23 ^a^	2.39 ± 0.23 ^b^	1.21 ± 0.14 ^c^
1-Hexanol-D	0.23 ± 0.01 ^e^	0.26 ± 0.01 ^e^	0.30 ± 0.00 ^d^	0.25 ± 0.02 ^e^	0.49 ± 0.03 ^a^	0.45 ± 0.01 ^b^	0.39 ± 0.03 ^c^
1-Hexanol-M	0.83 ± 0.03 ^d^	0.93 ± 0.02 ^c^	1.05 ± 0.01 ^b^	1.00 ± 0.02 ^b^	1.47 ± 0.02 ^a^	1.45 ± 0.04 ^a^	1.41 ± 0.06 ^a^
1-Pentanol-D	0.87 ± 0.06 ^a^	0.80 ± 0.08 ^a^	0.75 ± 0.05 ^a^	0.84 ± 0.09 ^a^	0.52 ± 0.05 ^b^	0.52 ± 0.07 ^b^	0.75 ± 0.05 ^a^
1-Pentanol-M	1.06 ± 0.02 ^c^	1.11 ± 0.04 ^c^	1.16 ± 0.04 ^bc^	1.23 ± 0.08 ^b^	1.09 ± 0.07 ^c^	1.13 ± 0.08 ^bc^	1.39 ± 0.07 ^a^
1-Penten-3-ol	0.75 ± 0.02 ^f^	0.82 ± 0.04 ^ef^	0.86 ± 0.03 ^de^	0.93 ± 0.06 ^cd^	1.00 ± 0.02 ^bc^	1.06 ± 0.06 ^b^	1.33 ± 0.05 ^a^
3-Methyl-1-butanol-D	0.23 ± 0.03 ^a^	0.23 ± 0.01 ^a^	0.26 ± 0.01 ^a^	0.21 ± 0.03 ^a^	0.35 ± 0.16 ^a^	0.30 ± 0.11 ^a^	0.23 ± 0.06 ^a^
3-Methyl-1-butanol-M	0.09 ± 0.06 ^c^	0.10 ± 0.07 ^bc^	0.13 ± 0.08 ^bc^	0.14 ± 0.07 ^bc^	0.24 ± 0.12 ^abc^	0.27 ± 0.14 ^ab^	0.38 ± 0.02 ^a^
Isobutanol	0.12 ± 0.01 ^d^	0.17 ± 0.00 ^bc^	0.20 ± 0.00 ^b^	0.19 ± 0.01 ^bc^	0.26 ± 0.03 ^a^	0.23 ± 0.03 ^a^	0.16 ± 0.01 ^c^
Total	6.28 ± 0.32 ^e^	6.57 ± 0.28 ^de^	7.00 ± 0.23 ^cd^	7.13 ± 0.45 b^cd^	8.13 ± 0.28 ^a^	7.79 ± 0.48 ^ab^	7.25 ± 0.34 ^abc^
**Aldehydes (15)**							
(E)-2-Heptenal-D	0.92 ± 0.03 ^a^	0.78 ± 0.01 ^b^	0.62 ± 0.01 ^c^	0.73 ± 0.06 ^b^	0.35 ± 0.05 ^e^	0.40 ± 0.06 ^e^	0.48 ± 0.03 ^d^
(E)-2-Heptenal-M	1.02 ± 0.02 ^c^	1.05 ± 0.01 ^c^	1.04 ± 0.02 ^c^	1.11 ± 0.02 ^b^	0.81 ± 0.04 ^e^	0.94 ± 0.05 ^d^	1.19 ± 0.03 ^a^
(E)-2-Octenal	1.88 ± 0.05 ^b^	1.76 ± 0.02 ^bc^	1.63 ± 0.05 ^c^	1.62 ± 0.05 ^c^	1.16 ± 0.08 ^d^	1.60 ± 0.05 ^c^	2.25 ± 0.28 ^a^
(E)-2-Pentenal-D	1.59 ± 0.06 ^a^	1.48 ± 0.02 ^ab^	1.41 ± 0.13 ^b^	1.45 ± 0.03 ^b^	0.97 ± 0.03 ^d^	1.10 ± 0.07 ^c^	1.38 ± 0.07 ^b^
(E)-2-Pentenal-M	0.57 ± 0.06 ^d^	0.65 ± 0.03 ^bc^	0.70 ± 0.03 ^b^	0.68 ± 0.04 ^bc^	0.62 ± 0.01 ^cd^	0.70 ± 0.03 ^b^	0.84 ± 0.04 ^a^
2-Hexenal-D	1.00 ± 0.17 ^c^	1.47 ± 0.12 ^b^	1.78 ± 0.11 ^b^	1.68 ± 0.15 ^b^	3.01 ± 0.35 ^a^	2.93 ± 0.39 ^a^	3.21 ± 0.08 ^a^
2-Hexenal-M	0.81 ± 0.05 ^e^	0.97 ± 0.03 ^d^	1.11 ± 0.04 ^cd^	1.14 ± 0.06 ^c^	1.42 ± 0.13 ^b^	1.48 ± 0.12 ^b^	1.73 ± 0.09 ^a^
3-Methyl-2-butenal	0.30 ± 0.01 ^bc^	0.31 ± 0.01 ^bc^	0.29 ± 0.01 ^cd^	0.34 ± 0.03 ^b^	0.19 ± 0.01 ^e^	0.25 ± 0.01 ^d^	0.43 ± 0.05 ^a^
3-Methylbutanal	0.53 ± 0.09 ^a^	0.56 ± 0.03 ^a^	0.51 ± 0.03 ^a^	0.56 ± 0.03 ^a^	0.34 ± 0.01 ^b^	0.41 ± 0.02 ^b^	0.58 ± 0.03 ^a^
Benzaldehyde-D	0.49 ± 0.03 ^ab^	0.46 ± 0.01 ^bc^	0.43 ± 0.01 ^c^	0.44 ± 0.01 ^c^	0.28 ± 0.02 ^e^	0.36 ± 0.02 ^d^	0.50 ± 0.04 ^a^
Benzaldehyde-M	3.31 ± 0.15 ^f^	3.55 ± 0.09 ^f^	4.06 ± 0.17 ^e^	4.47 ± 0.15 ^d^	5.28 ± 0.14 ^c^	5.81 ± 0.13 ^b^	7.42 ± 0.43 ^a^
Butanal	0.22 ± 0.05 ^c^	0.26 ± 0.01 ^bc^	0.29 ± 0.00 ^ab^	0.28 ± 0.03 ^b^	0.25 ± 0.02 b^c^	0.26 ± 0.03 ^bc^	0.33 ± 0.03 ^a^
Heptanal-D	0.65 ± 0.05 ^a^	0.54 ± 0.02 ^b^	0.44 ± 0.02 ^c^	0.54 ± 0.03 ^b^	0.16 ± 0.00 ^f^	0.23 ± 0.03 ^e^	0.34 ± 0.02 ^d^
Heptanal-M	0.68 ± 0.06 ^a^	0.70 ± 0.05 ^a^	0.70 ± 0.03 ^a^	0.75 ± 0.06 ^a^	0.50 ± 0.02 ^c^	0.59 ± 0.03 ^b^	0.76 ± 0.04 ^a^
Hexanal-D	0.65 ± 0.04 ^c^	0.68 ± 0.06 ^c^	0.66 ± 0.05 ^c^	0.78 ± 0.06 ^b^	0.51 ± 0.05 ^d^	0.67 ± 0.09 ^c^	1.00 ± 0.04 ^a^
Hexanal-M	0.52 ± 0.01 ^f^	0.58 ± 0.03 ^d^	0.64 ± 0.01 ^d^	0.70 ± 0.02 ^c^	0.68 ± 0.02 ^c^	0.79 ± 0.01 ^b^	1.06 ± 0.03 ^a^
Isobutanal	0.08 ± 0.05 ^b^	0.17 ± 0.01 ^a^	0.19 ± 0.01 ^a^	0.14 ± 0.05 ^ab^	0.12 ± 0.04 ^ab^	0.14 ± 0.06 ^ab^	0.14 ± 0.04 ^ab^
Methional-D	0.45 ± 0.01 ^a^	0.40 ± 0.01 ^b^	0.34 ± 0.01 ^c^	0.32 ± 0.01 ^c^	0.09 ± 0.01 ^e^	0.16 ± 0.01 ^d^	0.33 ± 0.04 ^c^
Methional-M	0.69 ± 0.02 ^bc^	0.70 ± 0.04 ^bc^	0.72 ± 0.02 ^b^	0.73 ± 0.02 ^b^	0.41 ± 0.01 ^d^	0.65 ± 0.02 ^c^	1.01 ± 0.06 ^a^
Pentanal	0.07 ± 0.02 ^d^	0.08 ± 0.01 ^cd^	0.09 ± 0.00 ^bc^	0.09 ± 0.01 ^bc^	0.08 ± 0.00 ^cd^	0.11 ± 0.01 ^ab^	0.12 ± 0.01 ^a^
Phenylacetaldehyde-D	0.48 ± 0.05 ^a^	0.41 ± 0.02 ^b^	0.36 ± 0.03 ^c^	0.33 ± 0.01 ^c^	0.18 ± 0.00 ^d^	0.18 ± 0.01 ^d^	0.21 ± 0.01 ^d^
Phenylacetaldehyde-M	1.91 ± 0.10 ^a^	1.88 ± 0.03 ^a^	1.77 ± 0.04 ^b^	1.78 ± 0.03 ^b^	0.87 ± 0.01 ^e^	1.20 ± 0.05 ^d^	1.51 ± 0.08 ^c^
Propanal	0.12 ± 0.02 ^a^	0.11 ± 0.02 ^a^	0.08 ± 0.00 ^a^	0.09 ± 0.03 ^a^	0.10 ± 0.03 ^a^	0.09 ± 0.03 ^a^	0.12 ± 0.04 ^a^
Total	18.95 ± 0.18 ^de^	19.55 ± 0.15 ^cd^	19.85 ± 0.39 ^c^	20.74 ± 0.18 ^b^	18.39 ± 0.67 ^e^	21.05 ± 0.64 ^b^	26.94 ± 0.65 ^a^
**Ketones (8)**							
1-Octen-3-one	0.24 ± 0.01 ^a^	0.21 ± 0.01 ^ab^	0.19 ± 0.00 ^ab^	0.17 ± 0.08 ^ab^	0.13 ± 0.05 ^b^	0.16 ± 0.05 ^ab^	0.19 ± 0.08 ^ab^
1-Penten-3-one-D	0.55 ± 0.27 ^c^	0.78 ± 0.04 ^ab^	0.72 ± 0.01 ^abc^	0.79 ± 0.09 ^ab^	0.68 ± 0.03 ^bc^	0.77 ± 0.07 ^ab^	0.94 ± 0.08 ^a^
1-Penten-3-one-M	0.21 ± 0.10 ^b^	0.31 ± 0.00 ^a^	0.32 ± 0.03 ^a^	0.33 ± 0.06 ^a^	0.34 ± 0.04 ^a^	0.37 ± 0.07 ^a^	0.41 ± 0.03 ^a^
2-Heptanone	0.09 ± 0.04 ^a^	0.09 ± 0.04 ^a^	0.09 ± 0.03 ^a^	0.10 ± 0.03 ^a^	0.10 ± 0.02 ^a^	0.10 ± 0.03 ^a^	0.13 ± 0.01 ^a^
3-Hydroxy-2-butanone	0.68 ± 0.01 ^e^	0.77 ± 0.07 ^de^	0.86 ± 0.05 ^d^	0.95 ± 0.09 ^cd^	1.12 ± 0.20 ^bc^	1.29 ± 0.11 ^b^	1.61 ± 0.02 ^a^
3-Hydroxy-2-butanone-D	1.09 ± 0.05 ^a^	0.95 ± 0.04 ^a^	0.95 ± 0.02 ^a^	0.93 ± 0.40 ^a^	0.71 ± 0.24 ^a^	0.81 ± 0.23 ^a^	1.18 ± 0.52 ^a^
3-Hydroxy-2-butanone-M	0.58 ± 0.02 ^c^	0.64 ± 0.00 ^bc^	0.72 ± 0.01 ^bc^	0.69 ± 0.12 ^bc^	0.79 ± 0.11 ^ab^	0.82 ± 0.14 ^ab^	0.95 ± 0.18 ^a^
6-Methylhept-5-en-2-one	0.26 ± 0.01 ^a^	0.24 ± 0.00 ^a^	0.26 ± 0.01 ^a^	0.22 ± 0.04 ^a^	0.21 ± 0.02 ^a^	0.24 ± 0.02 ^a^	0.23 ± 0.04 ^a^
Acetone	1.21 ± 0.06 ^d^	1.23 ± 0.01 ^d^	1.21 ± 0.08 ^d^	1.37 ± 0.07 ^c^	1.59 ± 0.07 ^b^	1.62 ± 0.02 ^b^	1.83 ± 0.08 ^a^
Cyclohexanone	0.83 ± 0.05 ^c^	0.85 ± 0.01 ^c^	0.86 ± 0.02 ^c^	0.89 ± 0.02 ^c^	0.86 ± 0.01 ^c^	1.00 ± 0.06 ^b^	1.35 ± 0.02 ^a^
Total	5.73 ± 0.49 ^c^	6.07 ± 0.07 ^c^	6.19 ± 0.04 ^c^	6.43 ± 0.62 ^bc^	6.55 ± 0.28 ^bc^	7.18 ± 0.59 ^b^	8.84 ± 0.85 ^a^
**Esters (15)**							
(Z)-3-Hexenyl acetate-D	0.74 ± 0.06 ^b^	0.70 ± 0.02 ^b^	0.70 ± 0.03 ^b^	0.65 ± 0.06 ^b^	0.95 ± 0.09 ^a^	0.90 ± 0.19 ^a^	1.00 ± 0.03 ^a^
(Z)-3-Hexenyl acetate-M	1.12 ± 0.16 ^d^	1.21 ± 0.11 ^cd^	1.34 ± 0.10 ^c^	1.38 ± 0.14 ^bc^	1.78 ± 0.09 ^a^	1.58 ± 0.12 ^b^	1.04 ± 0.02 ^d^
3-Methylbutyl 2-methylbutanoate	1.18 ± 0.08 ^a^	1.12 ± 0.02 ^a^	1.03 ± 0.04 ^b^	0.94 ± 0.07 ^b^	0.80 ± 0.04 ^c^	0.72 ± 0.03 ^d^	0.28 ± 0.02 ^e^
Butyl acetate	0.15 ± 0.03 ^a^	0.19 ± 0.02 ^a^	0.17 ± 0.01 ^a^	0.16 ± 0.03 ^a^	0.09 ± 0.01 ^b^	0.10 ± 0.02 ^b^	0.06 ± 0.03 ^b^
Butyl propionate-D	0.20 ± 0.04 ^a^	0.18 ± 0.02 ^a^	0.17 ± 0.03 ^a^	0.11 ± 0.04 ^b^	0.12 ± 0.04 ^b^	0.06 ± 0.02 ^bc^	0.02 ± 0.00 ^c^
Butyl propionate-M	0.16 ± 0.02 ^ab^	0.17 ± 0.00 ^ab^	0.18 ± 0.02 ^a^	0.15 ± 0.02 ^b^	0.17 ± 0.01 ^ab^	0.14 ± 0.02 ^b^	0.14 ± 0.00 ^b^
Dihydro-2(3H)-furanone	0.80 ± 0.05 ^a^	0.66 ± 0.01 ^b^	0.54 ± 0.00 ^c^	0.54 ± 0.02 ^c^	0.41 ± 0.01 ^d^	0.43 ± 0.03 ^d^	0.47 ± 0.06 ^d^
Ethyl acetate	1.18 ± 0.18 ^c^	1.5 ± 0.01 ^b^	1.49 ± 0.02 ^b^	1.52 ± 0.09 ^b^	1.94 ± 0.10 ^a^	1.93 ± 0.14 ^a^	2.06 ± 0.15 ^a^
Ethyl butanoate	0.28 ± 0.15 ^b^	0.45 ± 0.02 ^a^	0.36 ± 0.01 ^ab^	0.34 ± 0.04 ^b^	0.13 ± 0.01 ^c^	0.13 ± 0.01 ^c^	0.04 ± 0.00 ^c^
Hexyl 2-methylbutanoate-D	4.88 ± 0.24 ^a^	4.25 ± 0.01 ^b^	3.92 ± 0.01 ^c^	3.33 ± 0.17 ^e^	2.86 ± 0.14 ^e^	1.46 ± 0.23 ^f^	0.18 ± 0.01 ^g^
Hexyl 2-methylbutanoate-M	2.11 ± 0.05 ^c^	2.17 ± 0.04 ^bc^	2.33 ± 0.05 ^a^	2.32 ± 0.08 ^a^	2.26 ± 0.09 ^ab^	1.69 ± 0.05 ^d^	0.22 ± 0.04 ^e^
Hexyl isobutyrate	1.89 ± 0.11 ^a^	1.80 ± 0.02 ^a^	1.85 ± 0.03 ^a^	1.64 ± 0.03 ^b^	1.63 ± 0.06 ^b^	1.19 ± 0.03 ^c^	0.46 ± 0.08 ^d^
Isoamyl hexanoate-D	4.32 ± 0.21 ^a^	3.67 ± 0.09 ^b^	3.35 ± 0.03 ^c^	2.82 ± 0.05 ^d^	2.20 ± 0.05 ^e^	1.23 ± 0.16 ^f^	0.50 ± 0.07 ^g^
Isoamyl hexanoate-M	2.58 ± 0.10 ^bc^	2.57 ± 0.10 ^c^	2.79 ± 0.08 ^a^	2.74 ± 0.07 ^ab^	2.68 ± 0.10 ^abc^	2.00 ± 0.10 ^d^	0.29 ± 0.02 ^e^
Isoamyl isovalerate-D	4.17 ± 0.09 ^a^	3.65 ± 0.09 ^b^	3.36 ± 0.14 ^c^	2.7 ± 0.10 ^d^	2.37 ± 0.15 ^e^	1.33 ± 0.08 ^f^	0.53 ± 0.03 ^g^
Isoamyl isovalerate-M	1.41 ± 0.04 ^c^	1.59 ± 0.00 ^bc^	1.71 ± 0.01 ^b^	1.72 ± 0.09 ^b^	1.92 ± 0.04 ^a^	1.68 ± 0.03 ^b^	0.69 ± 0.26 ^d^
Linalyl acetate	3.79 ± 0.09 ^a^	3.36 ± 0.02 ^b^	2.98 ± 0.05 ^c^	2.78 ± 0.06 ^d^	2.64 ± 0.03 ^e^	2.04 ± 0.08 ^f^	1.24 ± 0.06 ^g^
Methyl hexanoate	0.70 ± 0.00 ^ab^	0.68 ± 0.03 ^ab^	0.71 ± 0.05 ^a^	0.59 ± 0.06 ^bc^	0.69 ± 0.11 ^ab^	0.49 ± 0.07 ^c^	0.10 ± 0.01 ^d^
Propyl acetate-D	0.04 ± 0.01 ^a^	0.04 ± 0.00 ^ab^	0.04 ± 0.00 ^ab^	0.03 ± 0.01 ^b^	0.04 ± 0.00 ^ab^	0.03 ± 0.01 ^ab^	0.03 ± 0.01 ^b^
Propyl acetate-M	0.38 ± 0.06 ^ab^	0.43 ± 0.01 ^a^	0.43 ± 0.01 ^a^	0.40 ± 0.06 ^a^	0.26 ± 0.07 ^b^	0.36 ± 0.09 ^ab^	0.44 ± 0.09 ^a^
γ-Butyrolactone-D	0.86 ± 0.06 ^a^	0.63 ± 0.02 ^b^	0.52 ± 0.03 ^c^	0.48 ± 0.03 ^c^	0.39 ± 0.01 ^d^	0.48 ± 0.03 ^c^	0.61 ± 0.03 ^b^
γ-Butyrolactone-M	1.64 ± 0.07 ^b^	1.48 ± 0.04 ^c^	1.41 ± 0.04 ^c^	1.38 ± 0.01 ^cd^	1.3 ± 0.02 ^d^	1.58 ± 0.06 ^b^	1.94 ± 0.09 ^a^
Total	34.58 ± 0.61 ^a^	32.49 ± 0.27 ^b^	31.35 ± 0.24 ^c^	28.72 ± 0.21 ^d^	27.63 ± 0.13 ^e^	21.56 ± 0.59 ^f^	12.37 ± 0.53 ^g^
**Acids (4)**							
Acetic acid	13.4 ± 0.09 ^f^	14.16 ± 0.27 ^ef^	15.04 ± 0.21 ^de^	15.77 ± 0.40 ^d^	17.09 ± 0.42 ^c^	19.3 ± 0.78 ^b^	23.65 ± 1.22 ^a^
Butanoic acid-D	5.04 ± 0.71 ^ab^	5.12 ± 0.09 ^a^	4.27 ± 0.31 ^bc^	4.49 ± 0.23 ^abc^	4.17 ± 0.16 ^c^	4.59 ± 0.19 ^abc^	3.98 ± 0.75 ^c^
Butanoic acid-M	6.37 ± 0.16 ^e^	6.90 ± 0.09 ^d^	7.00 ± 0.15 ^cd^	7.22 ± 0.08 ^c^	7.64 ± 0.17 ^b^	7.95 ± 0.21 ^a^	7.60 ± 0.24 ^b^
Isobutanoic acid-D	1.68 ± 0.20 ^b^	1.59 ± 0.04 ^b^	1.51 ± 0.11 ^b^	1.72 ± 0.10 ^b^	1.87 ± 0.09 ^ab^	2.17 ± 0.05 ^a^	2.21 ± 0.45 ^a^
Isobutanoic acid-M	1.30 ± 0.06 ^e^	1.42 ± 0.01 ^de^	1.56 ± 0.03 ^d^	1.72 ± 0.01 ^c^	2.05 ± 0.09 ^b^	2.19 ± 0.03 ^ab^	2.22 ± 0.19 ^a^
Propanoic acid-D	0.24 ± 0.01 ^a^	0.21 ± 0.02 ^bc^	0.19 ± 0.01 ^d^	0.19 ± 0.01 ^d^	0.18 ± 0.01 ^d^	0.19 ± 0.01 ^cd^	0.21 ± 0.01 ^b^
Propanoic acid-M	0.67 ± 0.02 ^b^	0.67 ± 0.02 ^b^	0.68 ± 0.03 ^b^	0.68 ± 0.03 ^b^	0.70 ± 0.02 ^b^	0.74 ± 0.03 ^b^	0.87 ± 0.11 ^a^
Total	28.70 ± 1.06 ^e^	30.07 ± 0.43 ^de^	30.25 ± 0.54 ^de^	31.79 ± 0.26 ^cd^	33.71 ± 0.92 ^c^	37.13 ± 1.23 ^b^	40.74 ± 2.08 ^a^
**Terpenes (4)**							
Limonene	0.48 ± 0.09 ^a^	0.48 ± 0.13 ^a^	0.48 ± 0.16 ^a^	0.38 ± 0.19 ^a^	0.38 ± 0.23 ^a^	0.31 ± 0.21 ^ab^	0.07 ± 0.00 ^b^
α-Terpinene	0.29 ± 0.12 ^a^	0.23 ± 0.07 ^ab^	0.23 ± 0.05 ^ab^	0.23 ± 0.06 ^ab^	0.20 ± 0.06 ^ab^	0.17 ± 0.05 ^ab^	0.11 ± 0.01 ^b^
α-Terpinolene	0.21 ± 0.02 ^a^	0.18 ± 0.04 ^a^	0.16 ± 0.02 ^ab^	0.11 ± 0.06 ^bc^	0.10 ± 0.05 ^bc^	0.09 ± 0.04 ^c^	0.07 ± 0.01 ^c^
β-Pinene-D	0.97 ± 0.04 ^a^	0.89 ± 0.02 ^b^	0.84 ± 0.02 ^c^	0.74 ± 0.01 ^d^	0.75 ± 0.03 ^d^	0.51 ± 0.01 ^e^	0.20 ± 0.02 ^f^
β-Pinene-M	0.99 ± 0.26 ^a^	1.01 ± 0.22 ^a^	1.14 ± 0.16 ^a^	1.19 ± 0.22 ^a^	1.35 ± 0.28 ^a^	1.15 ± 0.24 ^a^	0.14 ± 0.01 ^b^
Total	3.26 ± 0.39 ^a^	2.8 ± 0.14 ^b^	2.85 ± 0.15 ^b^	2.65 ± 0.11 ^b^	2.78 ± 0.10 ^b^	2.23 ± 0.06 ^c^	0.59 ± 0.02 ^d^
**Heterocyclic compounds (3)**							
2,3-Dimethyl-5-ethylpyrazine	1.04 ± 0.01 ^e^	1.12 ± 0.06 ^de^	1.19 ± 0.02 ^cd^	1.22 ± 0.08 ^bcd^	1.28 ± 0.06 ^bc^	1.36 ± 0.04 ^b^	1.52 ± 0.14 ^a^
2,3-Dimethylpyrazine	0.28 ± 0.03 ^a^	0.26 ± 0.01 ^ab^	0.22 ± 0.01 ^c^	0.22 ± 0.02 ^c^	0.19 ± 0.01 ^c^	0.2 ± 0.02 ^c^	0.23 ± 0.03 ^bc^
5-Methylfurfural-D	0.17 ± 0.04 ^a^	0.12 ± 0.01 ^b^	0.10 ± 0.01 ^bc^	0.10 ± 0.00 ^bc^	0.09 ± 0.00 ^c^	0.09 ± 0.01 ^bc^	0.08 ± 0.00 ^c^
5-Methylfurfural-M	0.90 ± 0.10 ^b^	0.80 ± 0.02 ^c^	0.76 ± 0.04 ^c^	0.83 ± 0.02 ^bc^	0.91 ± 0.02 ^b^	1.01 ± 0.02 ^a^	1.03 ± 0.09 ^a^
Total	2.39 ± 0.12 ^cd^	2.29 ± 0.06 ^d^	2.27 ± 0.02 ^d^	2.36 ± 0.08 ^cd^	2.47 ± 0.04 ^c^	2.66 ± 0.04 ^b^	2.86 ± 0.09 ^a^
**Sulfides (1)**							
Dimethyl sulfide	0.06 ± 0.01 ^e^	0.10 ± 0.01 ^d^	0.14 ± 0.00 ^cd^	0.15 ± 0.02 ^c^	0.28 ± 0.04 ^b^	0.33 ± 0.03 ^a^	0.34 ± 0.03 ^a^
Total	0.06 ± 0.01 ^e^	0.10 ± 0.01 ^d^	0.14 ± 0.00 ^cd^	0.15 ± 0.02 ^c^	0.28 ± 0.04 ^b^	0.33 ± 0.03 ^a^	0.34 ± 0.03 ^a^

^1^ “-M”, “-D”, and”-T” denote monomer, dimer, and trimer, respectively. The experimental results are presented as mean ± standard deviation of triplicate analysis. The different letters show a significant difference between chili oil samples in a relative amount of VOCs according to Duncan’s multiple range test (*p* < 0.05).

**Table 6:** The ROAV of key VOCs in chili oil samples.

Key Compounds ^1^	Odor Threshold (mg/kg)	ROAV							Odor Description
		PA	PB	PC	PD	PE	PF	PG	
**Alcohols**									
1-Hexanol-M	0.4	0.06 ± 0.00	0.07 ± 0.00	0.07 ± 0.00	0.07 ± 0.00	0.18 ± 0.00	0.11 ± 0.01	0.07 ± 0.00	fruity, flower, green
3-Methyl-1-butanol-M	0.1	0.03 ± 0.02	0.03 ± 0.02	0.04 ± 0.02	0.04 ± 0.02	0.12 ± 0.02	0.08 ± 0.04	0.07 ± 0.01	burnt, malt, banana, fruity
(Z)-3-Hexenol	1.1	0.06 ± 0.00	0.06 ± 0.01	0.06 ± 0.00	0.06 ± 0.01	0.12 ± 0.01	0.07 ± 0.01	0.02 ± 0.00	grass, fresh, oily, green
3-Methyl-1-butanol-D	0.1	0.07 ± 0.01	0.07 ± 0.00	0.07 ± 0.00	0.06 ± 0.01	0.17 ± 0.03	0.09 ± 0.03	0.05 ± 0.01	burnt, malt, banana, fruity
1-Penten-3-ol	0.4	0.05 ± 0.00	0.06 ± 0.01	0.06 ± 0.00	0.06 ± 0.01	0.12 ± 0.00	0.08 ± 0.01	0.07 ± 0.00	butter, pungent, green, fruity
**Aldehydes**									
(E)-2-Pentenal-M	0.3	0.06 ± 0.01	0.06 ± 0.00	0.06 ± 0.01	0.06 ± 0.00	0.10 ± 0.00	0.07 ± 0.01	0.06 ± 0.00	strawberry, fruit, pungent, fruity
Phenylacetaldehyde-M	0.022	2.51 ± 0.07	2.44 ± 0.12	2.25 ± 0.02	2.2 ± 0.04	1.95 ± 0.01	1.68 ± 0.04	1.36 ± 0.02	cocoa, grapefruit, green, peanut
Phenylacetaldehyde-D	0.022	0.63 ± 0.04	0.53 ± 0.02	0.46 ± 0.03	0.41 ± 0.03	0.40 ± 0.00	0.26 ± 0.01	0.19 ± 0.01	cocoa, grapefruit, green, peanut
Benzaldehyde-M	0.06	1.59 ± 0.02	1.69 ± 0.12	1.89 ± 0.12	2.03 ± 0.11	4.34 ± 0.18	2.98 ± 0.07	2.45 ± 0.25	almond, sweet, burnt sugar
Benzaldehyde-D	0.06	0.23 ± 0.01	0.22 ± 0.01	0.20 ± 0.00	0.20 ± 0.00	0.23 ± 0.01	0.18 ± 0.01	0.16 ± 0.01	almond, sweet, burnt sugar
Methional-M	0.0002	100.00 ± 0.00	100.00 ± 0.00	100.00 ± 0.00	100.00 ± 0.00	100.00 ± 0.00	100.00 ± 0.00	100.00 ± 0.00	creamy, peanut, cooked potato, beef
Methional-D	0.0002	64.77 ± 0.42	56.40 ± 1.39	47.38 ± 0.18	43.71 ± 1.76	21.65 ± 1.31	24.64 ± 2.19	32.45 ± 2.55	creamy, peanut, cooked potato, beef
(E)-2-Octenal	0.004	13.60 ± 0.13	12.56 ± 0.60	11.39 ± 0.09	11.08 ± 0.09	14.30 ± 0.69	12.31 ± 0.77	11.07 ± 0.68	nut, fat, herbal, fresh
(E)-2-Heptenal-M	0.05	0.59 ± 0.03	0.60 ± 0.03	0.58 ± 0.01	0.61 ± 0.01	0.80 ± 0.03	0.58 ± 0.04	0.47 ± 0.03	fat, fresh, pungent, almond
(E)-2-Heptenal-D	0.05	0.53 ± 0.01	0.44 ± 0.02	0.35 ± 0.01	0.40 ± 0.04	0.35 ± 0.04	0.25 ± 0.04	0.19 ± 0.00	fat, fresh, pungent, almond
2-Hexenal-D	0.85	0.03 ± 0.01	0.05 ± 0.01	0.06 ± 0.00	0.05 ± 0.01	0.17 ± 0.01	0.11 ± 0.02	0.07 ± 0.00	aldehydic, almond, green
(E)-2-Pentenal-D	0.3	0.15 ± 0.00	0.14 ± 0.01	0.13 ± 0.02	0.13 ± 0.01	0.16 ± 0.00	0.11 ± 0.01	0.09 ± 0.00	strawberry, fruit, pungent, fruity
3-Methylbutanal	0.013	1.19 ± 0.23	1.22 ± 0.03	1.09 ± 0.09	1.18 ± 0.03	1.30 ± 0.06	0.98 ± 0.08	0.89 ± 0.08	chocolate, ethereal, malt, fatty, aldehydic
Isobutanal	0.0034	0.70 ± 0.48	1.46 ± 0.16	1.57 ± 0.03	1.08 ± 0.39	1.69 ± 0.47	1.28 ± 0.49	0.82 ± 0.22	malt, fresh, floral
Propanal	0.0094	0.37 ± 0.08	0.32 ± 0.05	0.22 ± 0.01	0.25 ± 0.08	0.53 ± 0.06	0.28 ± 0.09	0.25 ± 0.11	earthy, cocoa, pungent, nutty
Hexanal-M	0.075	0.20 ± 0.01	0.22 ± 0.02	0.24 ± 0.01	0.26 ± 0.01	0.45 ± 0.01	0.32 ± 0.02	0.28 ± 0.01	fatty, fruity, aldehydic, green
Hexanal-D	0.075	0.25 ± 0.01	0.26 ± 0.04	0.25 ± 0.02	0.29 ± 0.03	0.34 ± 0.01	0.28 ± 0.04	0.26 ± 0.01	fatty, fruity, aldehydic, green
**Ketones**									
1-Penten-3-one-M	0.0007	8.56 ± 4.23	12.68 ± 0.71	12.78 ± 1.00	12.65 ± 1.94	24.01 ± 1.31	16.48 ± 3.13	11.59 ± 0.75	garlic, onion, fish, pungent
1-Penten-3-one-D	0.0007	22.75 ± 11.72	31.73 ± 1.27	28.86 ± 0.38	30.94 ± 2.75	48.17 ± 0.49	33.72 ± 3.47	26.64 ± 0.89	garlic, onion, fish, pungent
1-Octen-3-one	0.01	0.70 ± 0.06	0.60 ± 0.07	0.54 ± 0.02	0.46 ± 0.20	0.66 ± 0.17	0.48 ± 0.15	0.38 ± 0.14	earthy, metal, musty, mushroom
**Esters**									
Ethyl butanoate	0.03	0.27 ± 0.15	0.43 ± 0.05	0.33 ± 0.01	0.31 ± 0.03	0.21 ± 0.02	0.13 ± 0.02	0.03 ± 0.00	pineapple, banana, fruit
Ethyl acetate	0.94	0.04 ± 0.01	0.05 ± 0.00	0.04 ± 0.00	0.04 ± 0.00	0.10 ± 0.00	0.06 ± 0.01	0.04 ± 0.00	pineapple, ethereal, sweet, anise, fruity
**Acids**									
Butanoic acid-M	0.14	1.32 ± 0.08	1.40 ± 0.06	1.40 ± 0.04	1.41 ± 0.04	2.69 ± 0.05	1.75 ± 0.05	1.08 ± 0.10	fruit, cheese, butter, sweat, acetic
Butanoic acid-D	0.14	1.04 ± 0.18	1.04 ± 0.05	0.85 ± 0.06	0.88 ± 0.06	1.47 ± 0.03	1.01 ± 0.01	0.57 ± 0.14	fruit, cheese, butter, sweat, acetic
Isobutanoic acid-M	0.19	0.20 ± 0.02	0.21 ± 0.01	0.23 ± 0.01	0.25 ± 0.01	0.53 ± 0.02	0.35 ± 0.01	0.23 ± 0.03	butter, strawberry, cheese
Isobutanoic acid-D	0.19	0.26 ± 0.04	0.24 ± 0.02	0.22 ± 0.02	0.25 ± 0.02	0.49 ± 0.02	0.35 ± 0.00	0.23 ± 0.06	butter, strawberry, cheese
Acetic acid	0.5	0.78 ± 0.02	0.81 ± 0.03	0.84 ± 0.01	0.86 ± 0.01	1.69 ± 0.05	1.19 ± 0.03	0.94 ± 0.09	sour, pungent, sharp, vinegar
**Sulfides**									
Dimethyl sulfide	0.0012	1.50 ± 0.18	2.48 ± 0.34	3.29 ± 0.18	3.38 ± 0.38	11.39 ± 1.04	8.46 ± 0.64	5.59 ± 0.65	sulfurous, cabbage, onion, green

^1^ “-M”, “-D”, and”-T” denote monomer, dimer, and trimer, respectively. The threshold values in vegetable oil are documented in the literature. The ROAV are presented as mean ± standard deviation.

**Table 7:** The mixed ratios of Shuanla and Erjingtiao paprika for the preparation of chili oil samples.

Mixed Ratios	PA	PB	PC	PD	PE	PF	PG
*m*_Shuanla_:*m*_Erjingtiao_	10:0	8:2	6:4	5:5	4:6	2:8	0:10

## Data Availability

Data are contained within this article and Supplementary Materials.

## Supplementary Materials

The following supporting information can be downloaded at: https://www.mdpi.com/article/10.3390/molecules29235767/s1, Table S1: Volatile organic compounds (VOCs) identified by GC–IMS in chili oil.

## References

[1] Mendes N.D., Goncalves E. (2020). The Role of Bioactive Components Found in Peppers. Trends Food Sci. Technol..

[2] Sinisgalli C., Vezza T., Diez-Echave P., Ostuni A., Faraone I., Hidalgo-Garcia L., Russo D., Armentano M.F., Garrido-Mesa J., Rodriguez-Cabezas M.E. (2021). The Beneficial Effects of Red Sun-Dried *Capsicum annuum* L. Cv Senise Extract with Antioxidant Properties in Experimental Obesity Are Associated with Modulation of the Intestinal Microbiota. Mol. Nutr. Food Res..

[3] Ic E. (2022). Quantitative Viscosity Determination in Irradiated Major Spices (Black Pepper, Cardamom, Cinnamon, Ginger, and Turmeric) by Using a Vibro Viscometer for Long-Term Storage. Food Control.

[4] Mendes N.D., Santos M.C.P., Santos M.C.B., Cameron L.C., Ferreira M.S.L., Goncalves E. (2019). Characterization of Pepper (*Capsicum baccatum*)—A Potential Functional Ingredient. LWT Food Sci. Technol..

[5] Ye Z., Shang Z., Zhang S., Li M., Zhang X., Ren H., Hu X., Yi J. (2022). Dynamic Analysis of Flavor Properties and Microbial Communities in Chinese Pickled Chili Pepper (*Capsicum frutescens* L.): A Typical Industrial-Scale Natural Fermentation Process. Food Res. Int..

[6] Deng M.H., Wen J.F., Zhu H.S., Zou X.X. (2009). The Hottest Pepper Variety in China. Genet. Resour. Crop Evol..

[7] Yang M.L., Huang J., Zhou R.Q., Jin Y., Wu C.D., Zhao N. (2023). Discriminating the Effects of Microbial Fortification on Quality of Pixian Doubanjiang-*Pei* Manufactured by Different Chili Varieties. Food Biosci..

[8] Peng Y., Xu Y.H., Gao J.Y., Li M., Wen X., Ni Y.Y. (2024). Quality Characteristics of a Red Chili Oil Product Processed Via Oil Infusion: Effects of Pre-Heated Oil Temperature. J. Food Meas. Charact..

[9] Liu M., Hu L., Deng N., Cai Y., Li H., Zhang B., Wang J. (2024). Effects of Different Hot-Air Drying Methods on the Dynamic Changes in Color, Nutrient and Aroma Quality of Three Chili Pepper (*Capsicum annuum* L.) Varieties. Food Chem. X.

[10] Li D., Chu B., Li B., Wang X., Chen X., Gu Q. (2024). The Difference Analysis of Physicochemical Indexes and Volatile Flavor Compounds of Chili Oil Prepared from Different Varieties of Chili Pepper. Food Res. Int..

[11] Zhu Y., Li X., Jiang S., Zhang Y., Zhang L., Liu Y. (2023). Multi-Dimensional Pungency and Sensory Profiles of Powder and Oil of Seven Chili Peppers Based on Descriptive Analysis and Scoville Heat Units. Food Chem..

[12] Caporaso N., Paduano A., Nicoletti G., Sacchi R. (2013). Capsaicinoids, Antioxidant Activity, and Volatile Compounds in Olive Oil Flavored with Dried Chili Pepper (*Capsicum annuum*). Eur. J. Lipid Sci. Technol..

[13] Wang S.Q., Chen H.T., Sun B.G. (2020). Recent Progress in Food Flavor Analysis Using Gas Chromatography-Ion Mobility Spectrometry (GC-IMS). Food Chem..

[14] Hu X., Wang R., Guo J., Ge K., Li G., Fu F., Ding S., Shan Y. (2019). Changes in the Volatile Components of Candied Kumquats in Different Processing Methodologies with Headspace-Gas Chromatography-Ion Mobility Spectrometry. Molecules.

[15] Liu J.Q., Han L.J., Han W.Z., Gui L.S., Yuan Z.Z., Hou S.Z., Wang Z.Y., Yang B.C., Raza S.H.A., Alowais A.F.S. (2023). Effect of Different Heat Treatments on the Quality and Flavor Compounds of Black Tibetan Sheep Meat by HS-GC-IMS Coupled with Multivariate Analysis. Molecules.

[16] Liu M., Yang Y., Zhao X., Wang Y., Li M., Wang Y., Tian M., Zhou J. (2024). Classification and Characterization on Sorghums Based on HS-GC-IMS Combined with OPLS-DA and GA-PlS. Curr. Res. Food. Sci..

[17] Dang D.S., Buhler J.F., Stafford C.D., Taylor M.J., Shippen J.E., Dai X., England E.M., Matarneh S.K. (2021). Nix Pro 2 and Color Muse as Potential Colorimeters for Evaluating Color in Foods. LWT Food Sci. Technol..

[18] Nishino A., Yasui H., Maoka T. (2016). Reaction of Paprika Carotenoids, Capsanthin and Capsorubin, with Reactive Oxygen Species. J. Agric. Food Chem..

[19] Minguez-Mosquera M.I., Hornero-Mendez D. (1993). Separation and Quantification of the Carotenoid Pigments in Red Peppers (*Capsicum annuum* L.), Paprika, and Oleoresin by Reversed-Phase HPLC. J. Agric. Food Chem..

[20] Pereira E., Ferreira M.C., Sampaio K.A., Grimaldi R., Meirelles A.J.D.A., Maximo G.J. (2019). Physical Properties of Amazonian Fats and Oils and Their Blends. Food Chem..

[21] Asadi Y., Farahmandfar R. (2020). Frying Stability of Canola Oil Supplemented with Ultrasound-Assisted Extraction of Teucrium Polium. Food Sci. Nutr..

[22] Farhoosh R., Kenari R.E., Poorazrang H. (2009). Frying Stability of Canola Oil Blended with Palm Olein, Olive, and Corn Oils. J. Am. Oil Chem. Soc..

[23] Matsufuji H., Nakamura H., Chino M., Takeda M. (1998). Antioxidant Activity of Capsanthin and the Fatty Acid Esters in Paprika (*Capsicum annuum*). J. Agric. Food Chem..

[24] Cavazza A., Corti S., Mancinelli C., Bignardi C., Corradini C. (2015). Effect of the Addition of Chili Pepper Powder on Vegetable Oils Oxidative Stability. J. Am. Oil Chem. Soc..

[25] Chopan M., Littenberg B. (2017). The Association of Hot Red Chili Pepper Consumption and Mortality: A Large Population-Based Cohort Study. PLoS ONE.

[26] Guzmán I., Bosland P.W. (2017). Sensory Properties of Chile Pepper Heat–and Its Importance to Food Quality and Cultural Preference. Appetite.

[27] Zhang R., Chen K., Chen X., Yang B., Kan J. (2021). Thermostability and Kinetics Analysis of Oil Color, Carotenoids and Capsaicinoids in Hotpot Oil Models (Butter, Rapeseed Oil, and Their Blends). LWT.

[28] Cheok C.Y., Sobhi B., Mohd Adzahan N., Bakar J., Abdul Rahman R., Ab Karim M.S., Ghazali Z. (2017). Physicochemical Properties and Volatile Profile of Chili Shrimp Paste as Affected by Irradiation and Heat. Food Chem..

[29] Yang L.Z., Liu J., Wang X.Y., Wang R.R., Ren F., Zhang Q., Shan Y., Ding S.H. (2019). Characterization of Volatile Component Changes in Jujube Fruits During Cold Storage by Using Headspace-Gas Chromatography-Ion Mobility Spectrometry. Molecules.

[30] Lantsuzskaya E.V., Krisilov A.V., Levina A.M. (2015). Structure of the Cluster Ions of Ketones in the Gas Phase According to Ion Mobility Spectrometry and Ab Initio Calculations. Russ. J. Phys. Chem. A.

[31] Wu J., Chen X., Chen B., Pan N., Qiao K., Wu G., Liu Z. (2021). Collaborative Analysis Combining Headspace-Gas Chromatography-Ion Mobility Spectrometry (HS-GC-IMS) and Intelligent (Electronic) Sensory Systems to Evaluate Differences in the Flavour of Cultured Pufferfish. Flavour Fragr. J..

[32] Xu Y., Fan W., Qian M.C. (2007). Characterization of Aroma Compounds in Apple Cider Using Solvent-Assisted Flavor Evaporation and Headspace Solid-Phase Microextraction. J. Agric. Food Chem..

[33] Xu L., Mei X., Chang J., Wu G., Zhang H., Jin Q., Wang X. (2022). Comparative Characterization of Key Odorants of French Fries and Oils at the Break-in, Optimum, and Degrading Frying Stages. Food Chem..

[34] Zhang Q., Wan C., Wang C., Chen H., Liu Y., Li S., Lin D., Wu D., Qin W. (2018). Evaluation of the Non-Aldehyde Volatile Compounds Formed During Deep-Fat Frying Process. Food Chem..

[35] Jiang Y., Li D., Tu J., Zhong Y., Zhang D., Wang Z., Tao X. (2021). Mechanisms of Change in Gel Water-Holding Capacity of Myofibrillar Proteins Affected by Lipid Oxidation: The Role of Protein Unfolding and Cross-Linking. Food Chem..

[36] Mottram D.S. (1998). Flavour Formation in Meat and Meat Products: A Review. Food Chem..

[37] Gracka A., Jelen H.H., Majcher M., Siger A., Kaczmarek A. (2016). Flavoromics Approach in Monitoring Changes in Volatile Compounds of Virgin Rapeseed Oil Caused by Seed Roasting. J. Chromatogr. A.

[38] Huang Y.R., Tippmann J., Becker T. (2016). Non-Isothermal Kinetic Models of Degradation of S-Methylmethionine. J. Food Process Eng..

[39] Wu Z., Chen L., Wu L., Xue X., Zhao J., Li Y., Ye Z., Lin G. (2015). Classification of Chinese Honeys According to Their Floral Origins Using Elemental and Stable Isotopic Compositions. J. Agric. Food Chem..

[40] Kang C.D., Zhang Y.Y., Zhang M.Y., Qi J., Zhao W.T., Gu J., Guo W.P., Li Y.Y. (2022). Screening of Specific Quantitative Peptides of Beef by Lc-Ms/Ms Coupled with OPLS-DA. Food Chem..

[41] Yu J., Zhang Y., Wang Q., Yang L., Karrar E., Jin Q., Zhang H., Wu G., Wang X. (2023). Capsaicinoids and Volatile Flavor Compounds Profile of Sichuan Hotpot as Affected by Cultivar of Chili Peppers During Processing. Food Res. Int..

[42] Geng L.J., Zhou W., Qu X.Y., Sa R., Liang J., Wang X.Y., Sun M.Y. (2023). Iodine Values, Peroxide Values and Acid Values of Bohai Algae Oil Compared with Other Oils During the Cooking. Heliyon.

[43] Zhang N., Li Y.L., Wen S.S., Sun Y.W., Chen J., Gao Y., Sagymbek A., Yu X.Z. (2021). Analytical Methods for Determining the Peroxide Value of Edible Oils: A Mini-Review. Food Chem..

[44] Zhang Q., Tang J.N., Deng J., Cai Z.J., Jiang X.L., Zhu C.L. (2024). Effect of Capsaicin Stress on Aroma-Producing Properties of Lactobacillus Plantarum Cl-01 Based on E-Nose and GC-IMS. Molecules.

[45] Zhu Y.F., Chen J., Chen X.J., Chen D.Z., Deng S.G. (2020). Use of Relative Odor Activity Value (Roav) to Link Aroma Profiles to Volatile Compounds: Application to Fresh and Dried Eel (*Muraenesox cinereus*). Int. J. Food Prop..

[46] Gemert L.J.V. (2011). Odour Thresholds: Compilations of Odour Threshold Values in Air, Water and Other Media.

