# Supplementary material for: Quality Characteristics and Volatile Components of Chili Oil Prepared from the Combination of Shuanla and Erjingtiao Peppers
Source: Molecules. 2024 Dec 6;29(23):5767. doi: 10.3390/molecules29235767 (PMC11643616; doi:10.3390/molecules29235767)
Supplement: Supplementary file 1 [file molecules-29-05767-s001.zip › molecules-3302294-supplementary.pdf]

Quality Characteristics and Volatile Components of Chili Oil Prepared from the Combination of Shuanla and Erjingtiao Peppers

Fang Yang [1,*], Simin Yao [1], Haibin Yuan [2,3], Can Yuan [1,3] and Hongfeng Jia [1,3,*]

1 College of Culinary and Food Science Engineering, Sichuan Tourism University, Chengdu 610100, China; 18383515088@163.com (S.Y.); yuancan2019@sctu.edu.cn (C.Y.)
2 Faculty of Food and Biological Engineering, Chengdu University, Chengdu 610106, China; yuanhaibin@stu.cdu.edu.cn
3 Cuisine Science Key Laboratory of Sichuan Province, Sichuan Tourism University, Chengdu 610100, China
* Correspondence: sichuanyangf@sctu.edu.cn (F.Y.); jiahongfeng_cq@sctu.edu.cn (H.J.); Tel.: +86-028-84820244 (H.J.)

Supporting Information

Table S1. Volatile organic compounds (VOCs) identified by GC–IMS in chili oil

Compounds	CAS#	RI[a]	Rt[b] (sec)	Dt[c] (RIP Relative)
**Alcohols**				
(Z)-3-Hexenol	928-96-1	1385.3	952.190	1.50337
1-Hexanol-D	111-27-3	1352.7	861.091	1.63760
1-Hexanol-M	111-27-3	1353.2	862.492	1.32522
1-Pentanol-D	71-41-0	1235.1	580.268	1.50786
1-Pentanol-M	71-41-0	1236.0	582.223	1.25115
1-Penten-3-ol	616-25-1	1137.4	425.913	0.94439
3-Methyl-1-butanol-D	123-51-3	1191.8	494.045	1.49274
3-Methyl-1-butanol-M	123-51-3	1195.0	500.301	1.24628
Isobutanol	78-83-1	1068.8	356.598	1.17178
**Aldehydes**				
(E)-2-Heptenal-D	18829-55-5	1321.0	772.616	1.66345
(E)-2-Heptenal-M	18829-55-5	1321.0	772.616	1.25504
(E)-2-Octenal	2548-87-0	1466.8	1179.939	1.33254
(E)-2-Pentenal-D	1576-87-0	1126.5	412.289	1.35985
(E)-2-Pentenal-M	1576-87-0	1126.2	411.921	1.10738
2-Hexenal-D	505-57-7	1215.3	540.699	1.51414
2-Hexenal-M	505-57-7	1215.6	541.452	1.17720
3-Methyl-2-butenal	107-86-8	1195.2	500.653	1.08968
3-Methylbutanal	590-86-3	932.7	285.018	1.18219
Benzaldehyde-D	100-52-7	1464.3	1172.955	1.47344
Benzaldehyde-M	100-52-7	1469.5	1187.367	1.15854
Butanal	123-72-8	880.0	265.721	1.11312
Heptanal-D	111-71-7	1178.5	477.384	1.69345
Heptanal-M	111-71-7	1178.7	477.595	1.33152

Hexanal-D	66-25-1	1080.4	364.359	1.56224
Hexanal-M	66-25-1	1082.2	365.540	1.26567
Isobutanal	78-84-2	820.7	243.999	1.28105
Methional-D	3268-49-3	1455.3	1147.818	1.39652
Methional-M	3268-49-3	1454.7	1146.036	1.08902
Pentanal	110-62-3	980.4	302.508	1.18250
Phenylacetaldehyde-D	122-78-1	1672.9	1755.727	1.52606
Phenylacetaldehyde-M	122-78-1	1673.1	1756.362	1.25746
Propanal	123-38-6	802.2	237.235	1.04108
**Ketones**				
1-Octen-3-one	4312-99-6	1278.4	666.791	1.27417
1-Penten-3-one-D	1629-58-9	1015.9	321.462	1.30977
1-Penten-3-one-M	1629-58-9	1016.7	321.974	1.07868
2-Heptanone	110-43-0	1173.0	470.444	1.25940
3-Hydroxy-2-butanone	513-86-0	1301.8	718.806	1.05737
3-Hydroxy-2-butanone-D	513-86-0	1278.1	666.099	1.33097
3-Hydroxy-2-butanone-M	513-86-0	1280.2	670.356	1.06484
6-Methylhept-5-en-2-one	110-93-0	1332.9	805.775	1.17398
Acetone	67-64-1	831.8	248.072	1.11692
Cyclohexanone	108-94-1	1300.5	715.322	1.15106
**Acids**				
Acetic acid	64-19-7	1473.2	1197.901	1.05396
Butanoic acid-D	107-92-6	1610.2	1580.632	1.36750
Butanoic acid-M	107-92-6	1613.6	1589.917	1.15903
Isobutanoic acid-D	79-31-2	1554.2	1424.072	1.36453
Isobutanoic acid-M	79-31-2	1554.0	1423.514	1.14362
Propanoic acid-D	29102	1561.0	1443.054	1.26661
Propanoic acid-M	29102	1561.2	1443.612	1.11376
**Esters**				
(Z)-3-Hexenyl acetate-D	1708-82-3	1316.2	759.048	1.81329
(Z)-3-Hexenyl acetate-M	1708-82-3	1311.1	744.924	1.31962
3-Methylbutyl 2-methylbutanoate	27625-35-0	1304.9	727.441	1.42543
Butyl acetate	123-86-4	1044.1	340.182	1.23195
Butyl propionate-D	590-01-2	1143.9	434.045	1.70627
Butyl propionate-M	590-01-2	1143.7	433.847	1.28474
Dihydro-2(3H)-furanone	96-48-0	1591.8	1529.247	1.08959
Ethyl acetate	141-78-6	871.6	262.636	1.33653
Ethyl butanoate	105-54-4	1036.1	334.866	1.20030
Hexyl 2-methylbutanoate-D	10032-15-2	1427.9	1071.320	2.16474
Hexyl 2-methylbutanoate-M	10032-15-2	1427.2	1069.218	1.52534
Hexyl isobutyrate	2349-07-7	1339.3	823.741	1.46448
Isoamyl hexanoate-D	2198-61-0	1448.6	1129.158	2.13952
Isoamyl hexanoate-M	2198-61-0	1449.1	1130.379	1.51710

Isoamyl isovalerate-D	659-70-1	1287.2	684.282	2.05736
Isoamyl isovalerate-M	659-70-1	1290.1	690.188	1.45664
Linalyl acetate	115-95-7	1567.1	1460.165	1.21885
Methyl hexanoate	106-70-7	1191.4	493.487	1.29392
Propyl acetate-D	109-60-4	954.5	293.028	1.47648
Propyl acetate-M	109-60-4	956.8	293.874	1.16428
γ-Butyrolactone-D	96-48-0	1634.9	1649.609	1.30584
γ-Butyrolactone-M	96-48-0	1636.8	1654.915	1.08563
**Terpenes**				
Limonene	138-86-3	1186.4	487.329	1.22016
α-Terpinene	99-86-5	1169.4	465.981	1.21444
α-Terpinolene	586-62-9	1268.7	647.477	1.22089
β-Pinene-D	127-91-3	1109.2	390.600	1.21861
β-Pinene-M	127-91-3	1109.2	390.600	1.63364
**Heterocyclic compounds**				
2,3-Dimethyl-5-ethylpyrazine	15707-34-3	1466.5	1178.944	1.22396
2,3-Dimethylpyrazine	5910-89-4	1339.6	824.625	1.10416
5-Methylfurfural-D	620-02-0	1580.5	1497.685	1.47257
5-Methylfurfural-M	620-02-0	1580.9	1498.727	1.13289
**Sulfides**				
Dimethyl sulfide	75-18-3	805.3	238.361	0.96052

[a]RI : retention index; [b]Rt : retention time; [c]Dt: drift time.
